# Comparative Genomic and Phylogenetic Analyses of Gammaproteobacterial *glg* Genes Traced the Origin of the *Escherichia coli* Glycogen *glgBXCAP* Operon to the Last Common Ancestor of the Sister Orders *Enterobacteriales* and *Pasteurellales*

**DOI:** 10.1371/journal.pone.0115516

**Published:** 2015-01-21

**Authors:** Goizeder Almagro, Alejandro M. Viale, Manuel Montero, Mehdi Rahimpour, Francisco José Muñoz, Edurne Baroja-Fernández, Abdellatif Bahaji, Manuel Zúñiga, Fernando González-Candelas, Javier Pozueta-Romero

**Affiliations:** 1 Instituto de Agrobiotecnología (CSIC/UPNA/Gobierno de Navarra), Iruñako etorbidea 123, 31192 Mutiloabeti, Nafarroa, Spain; 2 Instituto de Biología Molecular y Celular de Rosario (IBR, CONICET), Departamento de Microbiología, Facultad de Ciencias Bioquímicas y Farmacéuticas, Universidad Nacional de Rosario (UNR), Suipacha 531, 2000 Rosario, Argentina; 3 Dpt. Biotecnología de Alimentos, Instituto de Agroquímica y Tecnología de Alimentos, CSIC, Calle Agustín Escardino, 7, 46980 Paterna, Valencia, Spain; 4 Unidad Mixta Genómica y Salud, FISABIO-Salud Pública/Instituto Cavanilles de Biodiversidad y Biología Evolutiva, Universidad de Valencia, Calle Catedrático José Beltrán Martínez, 246980 Paterna, Valencia, Spain; Wilfrid Laurier University, CANADA

## Abstract

Production of branched α-glucan, glycogen-like polymers is widely spread in the Bacteria domain. The glycogen pathway of synthesis and degradation has been fairly well characterized in the model enterobacterial species *Escherichia coli* (order *Enterobacteriales*, class *Gammaproteobacteria*), in which the cognate genes (branching enzyme *glgB*, debranching enzyme *glgX*, ADP-glucose pyrophosphorylase *glgC*, glycogen synthase *glgA*, and glycogen phosphorylase *glgP*) are clustered in a *glgBXCAP* operon arrangement. However, the evolutionary origin of this particular arrangement and of its constituent genes is unknown. Here, by using 265 complete gammaproteobacterial genomes we have carried out a comparative analysis of the presence, copy number and arrangement of *glg* genes in all lineages of the *Gammaproteobacteria*. These analyses revealed large variations in *glg* gene presence, copy number and arrangements among different gammaproteobacterial lineages. However, the *glgBXCAP* arrangement was remarkably conserved in all *glg*-possessing species of the orders *Enterobacteriales* and *Pasteurellales* (the E/P group). Subsequent phylogenetic analyses of *glg* genes present in the *Gammaproteobacteria* and in other main bacterial groups indicated that *glg* genes have undergone a complex evolutionary history in which horizontal gene transfer may have played an important role. These analyses also revealed that the E/P *glgBXCAP* genes (a) share a common evolutionary origin, (b) were vertically transmitted within the E/P group, and (c) are closely related to *glg* genes of some phylogenetically distant betaproteobacterial species. The overall data allowed tracing the origin of the *E. coli glgBXCAP* operon to the last common ancestor of the E/P group, and also to uncover a likely *glgBXCAP* transfer event from the E/P group to particular lineages of the *Betaproteobacteria*.

## Introduction

Production of large intracellular α-glucans composed of α-1,4-linked glucose units displaying low extents of α-1,6-linked branches (“bacterial glycogen”) represents a common feature observed in many groups of bacteria [1–9]. Glycogen constitutes a major carbon and energy reserve polymer that accumulates to cope with the starvation conditions often occurring in natural environments. The exact role of this polysaccharide in bacteria is not as clear-cut as in eukaryotes [7], but several studies have indicated that glycogen metabolism may confer some adaptive advantages including increased environmental survival and colonization and, in the case of pathogens, increased virulence and immune system evasion [1, 2, 8, 10–15].

**Figure 1:**
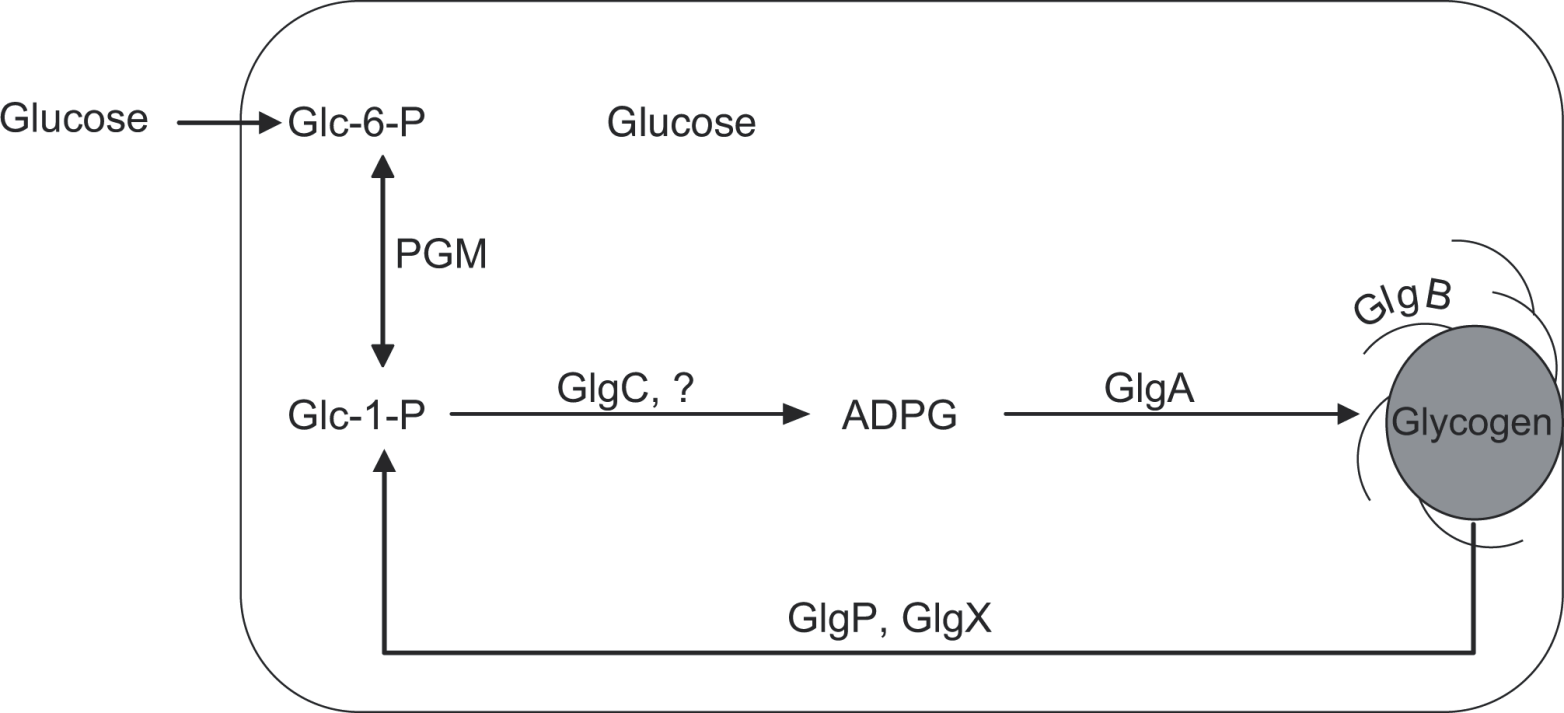
Schematic model of glycogen metabolism. Sugar (glucose) is incorporated into the cell and successively transformed to glucose-6-P (Glc-6-P), glucose-1-P (Glc-1-P), ADP-glucose (ADPG) and glycogen. This model involves the coupled reactions of phosphoglucomutase (PGM), ADPG pyrophosphorylase (GlgC) and other (?) ADPG-generating enzyme(s) [19,20], glycogen synthase (GlgA) and glycogen branching enzyme (GlgB). Glycogen catabolism is controlled by both glycogen phosphorylase (GlgP) and glycogen debranching enzyme (GlgX).

Studies conducted for more than 40 years in model bacterial species such as *Escherichia coli* and *Salmonella enterica* (family *Enterobacteriaceae*, order *Enterobacteriales*, class *Gammaproteobacteria*) were pivotal to elucidate the glycogen metabolic pathway schematically illustrated in **Fig. 1** [6,7]. According to this model, carbon sources are taken up by the bacterial cell and eventually transformed into glucose-6-phosphate (Glc-6-P), which is then converted into glucose-1-phosphate (Glc-1-P) by phosphoglucomutase (PGM). In the presence of Mg^2+^ and ATP, this hexose-P is converted into ADP-glucose (ADPG) and inorganic pyrophosphate by means of ADPG pyrophosphorylase (GlgC), the main regulatory step of glycogen synthesis [16]. ADPG is used by glycogen synthase (GlgA) to incorporate a new α-1,4-linked unit into the growing linear α-glucan. After chain elongation by GlgA, the glycogen branching enzyme (GlgB) catalyzes the formation of branched α-1,6-glycosidic linkages by transferring non-reducing-end oliglucans to the C-6-position of residues within a chain. On the catabolic side, the glycogen phosphorylase (GlgP) and glycogen debranching enzyme (GlgX) are the major determinants of glycogen degradation catalyzing the phosphorolytic cleavage of α-1,4 bonds to generate Glc-1-P at the non-reducing ends and the hydrolysis of α-1,6-glycosidic linkages on limit dextrins (the polysaccharide fragments remaining at the end of exhaustive hydrolysis of glycogen by GlgP), respectively [17,18]. This model is considered to represent the prevalent pathway of glycogen synthesis and degradation in bacteria [6,7]. Still, alternative glycogen synthesis pathways have been identified in some bacterial groups such as the high-G+C Gram-positive actinomycetes [12]. Also, yet unidentified sources of ADPG linked to glycogen biosynthesis have been described in both enterobacterial and cyanobacterial species [14,19,20].

Bacterial genes encoding functionally related proteins are generally clustered in operons [21–23]. This is the case of the five *E. coli glg* genes coding for the enzymes of the glycogen pathway, which are present in a single copy each and clustered in a single *glgBXCAP* operon arrangement [24]. However, the evolutionary history of this arrangement and its constituent genes remains unknown. To gain insight into the origin and evolutionary history of *E. coli* and *S. enterica glg* genes, we have carried out a detailed comparative analysis of the presence, copy number and arrangement of *glg* genes present in the different gammaproteobacterial lineages. Our analyses reveal the occurrence of large variations in *glg* homologs copy number and arrangements within the *Gammaproteobacteria, glgBXCAP* arrangement being highly conserved in all *glg* possessing species of the sister orders *Enterobacteriales* and *Pasteurellales* (the E/P group). Moreover, the phylogenetic analyses conducted in this work indicated that all *glg* genes in the E/P group share a common evolutionary origin. Finally, an extended analysis including *glg* genes of the main bacterial groups outside the *Gammaproteobacteria* indicates that E/P Glg proteins are more closely related to its counterparts of the phylogenetically distant betaproteobacterial species *Variovorax paradoxus* S110, *Thauera* sp. mz1t, *Leptothrix cholodnii* SP-6 and *Thiomonas intermedia* K12 than to Glg proteins of other *Gammaproteobacteria*. Furthermore, the genomic organization of *glg* genes in these betaproteobacterial species is very similar to that present in the E/P group.

## Results

### Identification and distribution of *glg* genes in the *Gammaproteobacteria*

We first searched for homologs of the five *glg* genes composing the *E. coli glgBXCAP* operon in entire genomes of the *Gammaproteobacteria* available in GenBank. The searches were conducted with the TBLASTN and PSI-BLAST programs on 265 complete gammaproteobacterial genomes representing 63 different genera and 13 orders using the GlgB, GlgX, GlgC, GlgA and GlgP protein sequences of the *E. coli* K-12 strain MG1655 (**S1 Table**) as query sequences. *Acidithiobacillales* were not included in this analysis since this proteobacterial order was recently classified as not belonging to the *Gammaproteobacteria* [25]. The number of sequenced genomes from the *Enterobacteriales* is over-represented in the databases as shown by the 115 out of the 265 genomes analyzed which belonged to species of this order **(S1 Table).**

**Figure 2:**
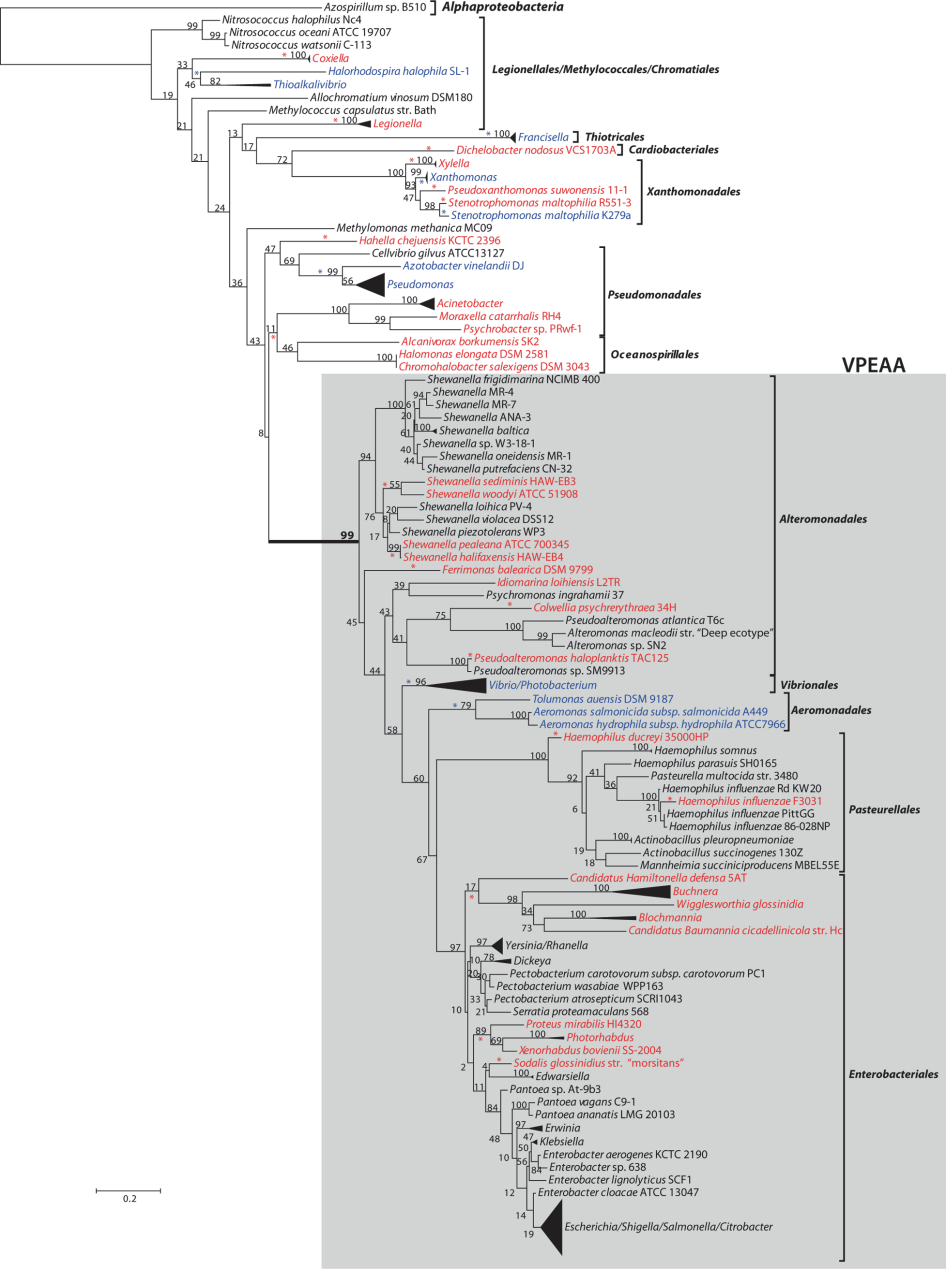
Summarized maximum-likelihood phylogenetic tree for 16S rRNA of the analyzed gammaproteobacterial species. Gammaproteobacterial orders are denoted by brackets. The tree was rooted with the alphaproteobacterial species *Azospirillum* sp. B510. Species with partial sets or total lack of *glg* genes are highlighted with blue and red colors, respectively. Support values for the bootstrap analysis by maximum likelihood are given. Red and blue asterisks highlight independent evolutionary events that result in total or partial *glg* losses, respectively. The complete tree is shown in S1 Fig.

Our analysis revealed a varied and complex distribution of *glg* genes in the *Gammaproteobacteria* (**S1 Table**). In general, the gammaproteobacterial species analyzed can be grouped into the following four categories: (i) organisms completely lacking *glg* homologs, (ii) organisms harbouring partial sets of *glg* homologs, (iii) organisms harbouring more than one copy of a particular *glg* homolog, and (iv) organisms harbouring all five *glg* homologs. This classification is not intended to be mutually exclusive since some species can be simultaneously included into categories (ii) and (iii), or (ii) and (iv) (**S1 Table**). Taking into account that most analyzed species are included in category (iv), in the following lines we will focus on categories (i), (ii) and (iii), and the results will be referenced to a phylogenetic tree based on 16S rRNA sequences (**Fig. 2; S1 Fig.**).

#### Gammaproteobacterial species lacking all five *glg* genes

Individual species or even complete groups in which no *glg* homologues could be identified (highlighted in red color in **Fig. 2**) are interspersed among the different gammaproteobacterial lineages. Among the most recently emerged lineages such as the *Enterobacteriales* [25,26], organisms lacking all *glg* genes include endosymbionts of insects or nematodes [27] of the genera *Buchnera, Blochmannia, Sodalis, Wigglesworthia* and *Photorhabdus*, as well as *Proteus mirabilis* HI4320, an inhabitant of the intestinal tract of humans and common cause of urinary infections [26] (**Fig. 2**).

In the order *Pasteurelalles*, sister clade of the *Enterobacteriales* [25,26], two phylogenetically distant species of *Haemophilus* (i. e., *H. influenzae* F3031 and *H. ducreyi*) completely lack *glg* genes, which contrasts with phylogenetically close relatives such as *H. influenzae* PittGG (**Fig. 2**).

Within the order *Alteromonodales* some species totally lack *glg* genes, such as the marine psychrophiles *Pseudoalteromonas haloplanktis* TAC125, *Idiomarina loihiensis* L2TR, and some species of the genus *Shewanella* including *S. woody* ATCC51908, *S. sedimis* HAW-E4, *S. pealeana* ANG-SQ1, *S. halifaxensis* HAW-E4, and *S. denitrificans* OS217 (**S1 Table; Fig. 2**). On the contrary, *glg* genes are present in other species of this order such as the marine *Alteromonas* isolates and the psychrophiles *Pseudoalteromonas atlantica* T6c and *Pseudoalteromonas sp.* SM9913 (**S1 Table; Fig. 2**).

Among the *Pseudomonadales*, organisms lacking *glg* genes include all the analyzed species of the genus *Acinetobacter* (encompassing both opportunistic human pathogens and harmless soil bacteria), the human pathogen *Moraxella catarrhalis* RH4, and *Psychrobacter* sp. PRwf-1 which inhabits extremely cold habitats (**S1 Table; Fig. 2**).

Finally, concerning the deepest diverging gammaproteobacterial branches, *glg* genes are totally absent in all analyzed species of the genera *Coxiella* (obligate intracellular pathogens) and *Legionella* (facultative intracellular pathogens), both assigned to the order *Legionellales*, and in *Dichelobacter nodosus* VCS1703A (aerotolerant anaerobic bacteria causing ovine foot rot) of the order *Cardiobacteriales* (**S1 Table; Fig. 2**). The same occurs in the genus *Xylella* (plant pathogens) and in *Stenotrophomonas maltophilia* R551-3 (human pathogen) both assigned to the order *Xanthomonadales.* Also *Alcanivorax borkumensis* SK2, *Hahella chejuensis* KCTC2396 and *Chromohalobacter salexigens* DSM3043 (order *Oceanospirillales*) inhabiting marine environments completely lack *glg* genes (**S1 Table; Fig. 2**).

#### Gammaproteobacterial species containing partial sets of *glg* genes

With the exception of *glgA* and *glgB*, differential absences of homologs of all other *glg* genes were observed in many independent lineages of the *Gammaproteobacteria* (in blue color in **Fig. 2**). For instance, among the order *Pseudomonadales,* all species of the genus *Pseudomonas* (which include human and plant pathogens) as well as *Azotobacter vinelandii* DJ (a nitrogen-fixer soil bacterium) contain homologs of all analyzed *glg* genes with the exception of *glgC* (**S1 Table; Fig. 2**). This is not the only example of species or groups totally lacking *glgC* homologs because, among the order *Xanthomonadales*, all the species of the genus *Xanthomonas* also lack *glgC* and, in addition, *glgP* (**S1 Table; Fig. 2**). Among the order *Vibrionales, Photobacterium profundum* SS9 and all the species of the genus *Vibrio* (including both marine species and human pathogens) lack *glgP*, but contain all other *glg* genes (**S1 Table; Fig. 2**). All analyzed species of the genus *Francisella* (order *Thiotricales*) lack *glgX* (**S1 Table; Fig. 2**). Finally, partial sets of *glg* genes are present in species assigned to the earliest emerging gammaproteobacterial lineages of the order *Chromatiales* including the phototrophic purple sulfur *Halorhodospira halophila* SL1, the ammonia-oxidizing chemolithoautotrophs of the genus *Nitrosococcus*, and the obligate haloalkaliphilic sulfur-oxidizing chemolithoautotroph *Thioalkalivibrio sulfidophilus* HL-EbGR7 (**S1 Table; Fig. 2**).

#### Gammaproteobacterial species possessing more than one copy of a particular *glg* homolog

Many gammaproteobacterial species display more than one homolog of a particular *glg* gene, a situation noted for all five *glg* genes (**S1 Table**). More than one homologous copy for a particular *glg* gene is observed at almost all branching levels, from the most recently emerged to the deepest ones (**Fig. 2**). Most notable examples in this context are provided by *Psychromonas ingrahamii* 37 (order *Alteromonadales*) which contains three *glgX* homologs, four *glgC* homologs, and two *glgA* homologs, besides one copy each of *glgB* and *glgP*, and *Allochromatium vinosum* DSM180 (order *Chromatiales*) with three copies of *glgA*, two each of *glgC, glgX* and *glgP*, besides one *glgB* copy (**S1 Table**). In turn, all the analyzed species of the genus *Xanthomonas* (order *Xanthomonadales*) possess two homologous copies of both *glgB* and *glgX* (**S1 Table**). Among the latest emerging gammaproteobacterial groups, all *Vibrio* species (order *Vibrionales*) possess two *glgC* homologs but only one copy of each of the other *glg* genes, and a similar situation was found in *Edwardsiella tarda* EIB202 of the order *Enterobacteriales* (**S1 Table**). Finally, in the deepest gammaproteobacterial branches (for example the order *Chromatiales*), *Nitrosococcus oceanii* ATCC19707 contains two *glgA* homologs whereas *H. halophila* SL1 contains two *glgC* homologs (**S1 Table**).

### Phylogenetic analysis of gammaproteobacterial Glg proteins

To determine the origin and evolutionary history of enterobacterial *glg* genes we conducted a phylogenetic analysis of Glg proteins in all analyzed gammaproteobacterial species. The data sets including Glg amino acid sequences were aligned using ClustalW and the alignments were subsequently refined with Gblocks. The resulting data sets consisted of 206 sequences with 537 conserved positions for the GlgB alignment, 202 sequences and 384 conserved positions for the GlgX alignment, 181 sequences and 331 conserved positions for the GlgC alignment, 201 sequences and 236 conserved positions for the GlgA alignment and 168 sequences and 631 conserved positions for the GlgP alignment.

ProtTest was used to determine the best-fit model of amino acid substitution. The LG+G+I+F model (see Materials and Methods) was identified as the best model for the five data sets. The phylogenetic information content of the data sets was then evaluated by using likelihood mapping. Briefly, this analysis allows to estimate the suitability for phylogenetic reconstruction of a data set from the proportion of unresolved quartets in a maximum likelihood analysis (for a complete description see Materials and Methods). The analysis was carried out using TreePuzzle with the WAG+G+F [28] model of substitution (the second best model selected by ProtTest since the LG model is not implemented in this program). The likelihood mapping showed that the five data sets possessed a high content of phylogenetic information, with 95%, 93%, 96%, 94% and 97% of fully resolved quartets in GlgB, GlgX, GlgC, GlgA and GlgP, respectively (**S2 Fig.**).

**Figure 3:**
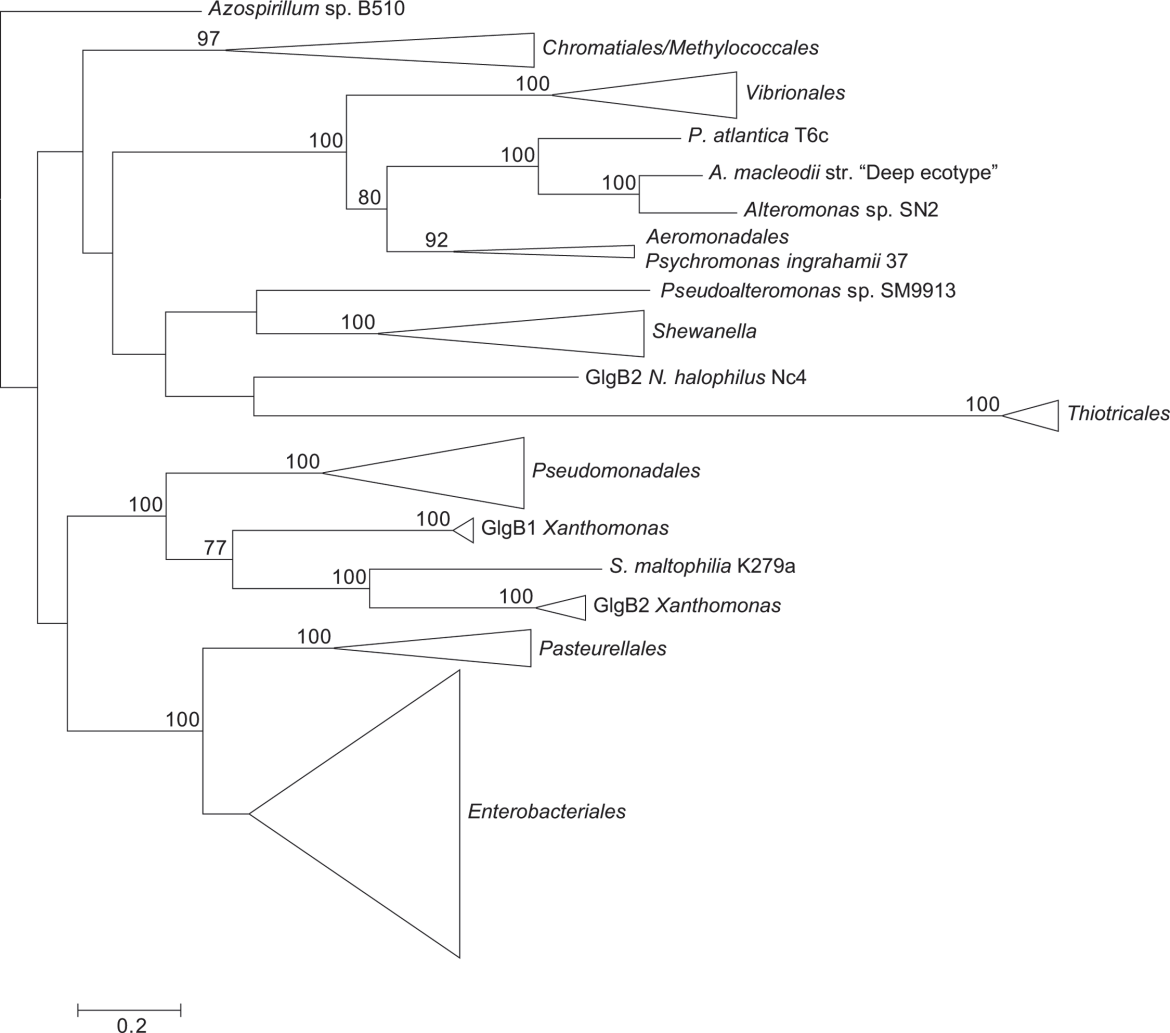
Summarized maximum-likelihood phylogenetic tree of the GlgB amino acid sequences containing all analyzed gammaproteobacterial species. The tree was arbitrarily rooted with the alphaproteobacterial species *Azospirillum* sp. B510. The complete tree is shown in S3 Fig. Bootstrap support values (>70%) are indicated.

**Figure 4:**
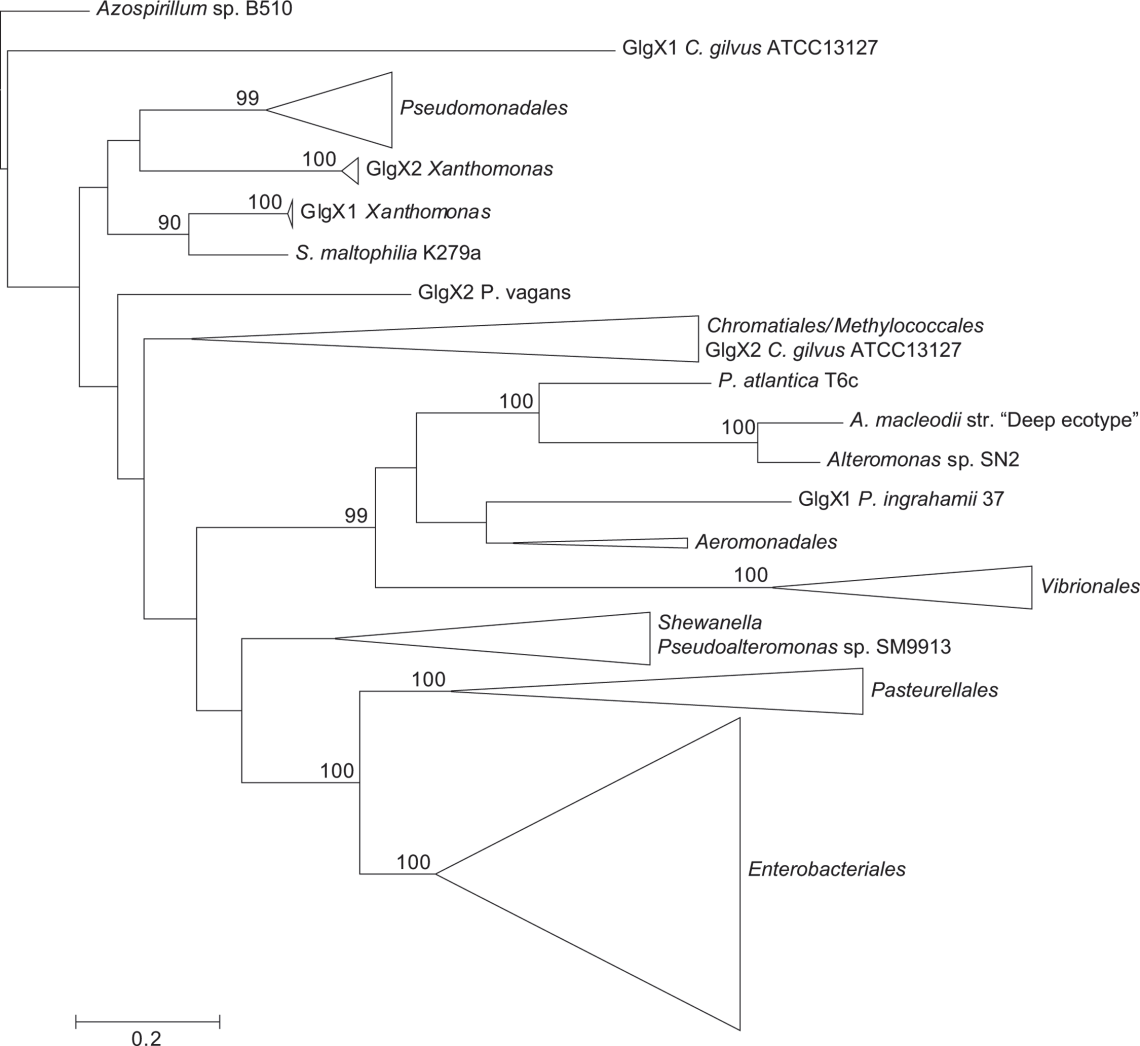
Summarized maximum-likelihood phylogenetic tree of the GlgX amino acid sequences containing all analyzed gammaproteobacterial species. The tree was arbitrarily rooted with the alphaproteobacterial species *Azospirillum* sp. B510. The complete tree is shown in S4 Fig. Bootstrap support values (>70%) are indicated.

**Figure 5:**
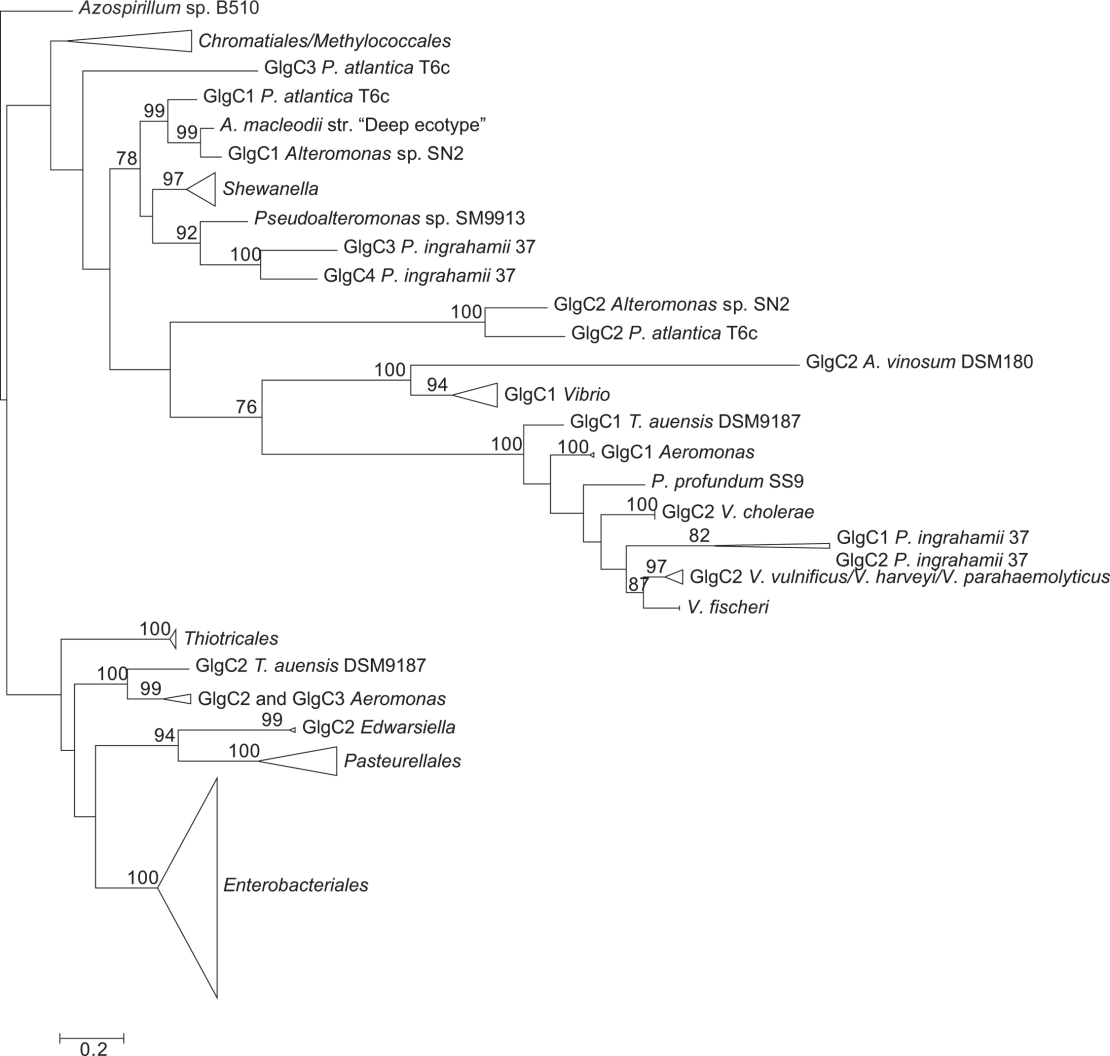
Summarized maximum-likelihood phylogenetic tree of the GlgC amino acid sequences containing all analyzed gammaproteobacterial species. The tree was arbitrarily rooted with the alphaproteobacterial species *Azospirillum* sp. B510. The complete tree is shown in S5 Fig. Bootstrap support values (>70%) are indicated.

**Figure 6:**
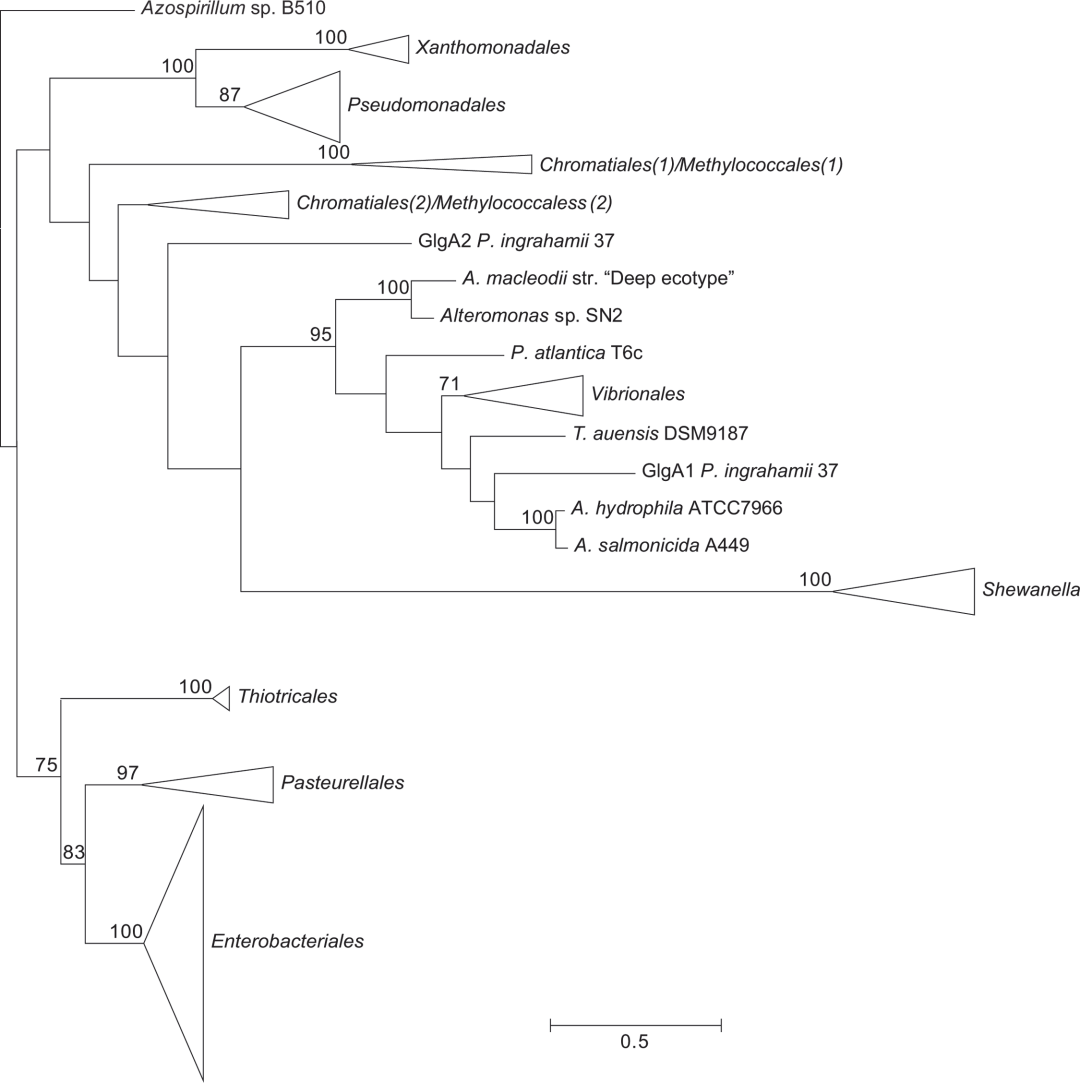
Summarized maximum-likelihood phylogenetic tree of the GlgA amino acid sequences containing all analyzed gammaproteobacterial species. The tree was arbitrarily rooted with the alphaproteobacterial species *Azospirillum* sp. B510. The complete tree is shown in S6 Fig. Bootstrap support values (>70%) are indicated.

**Figure 7:**
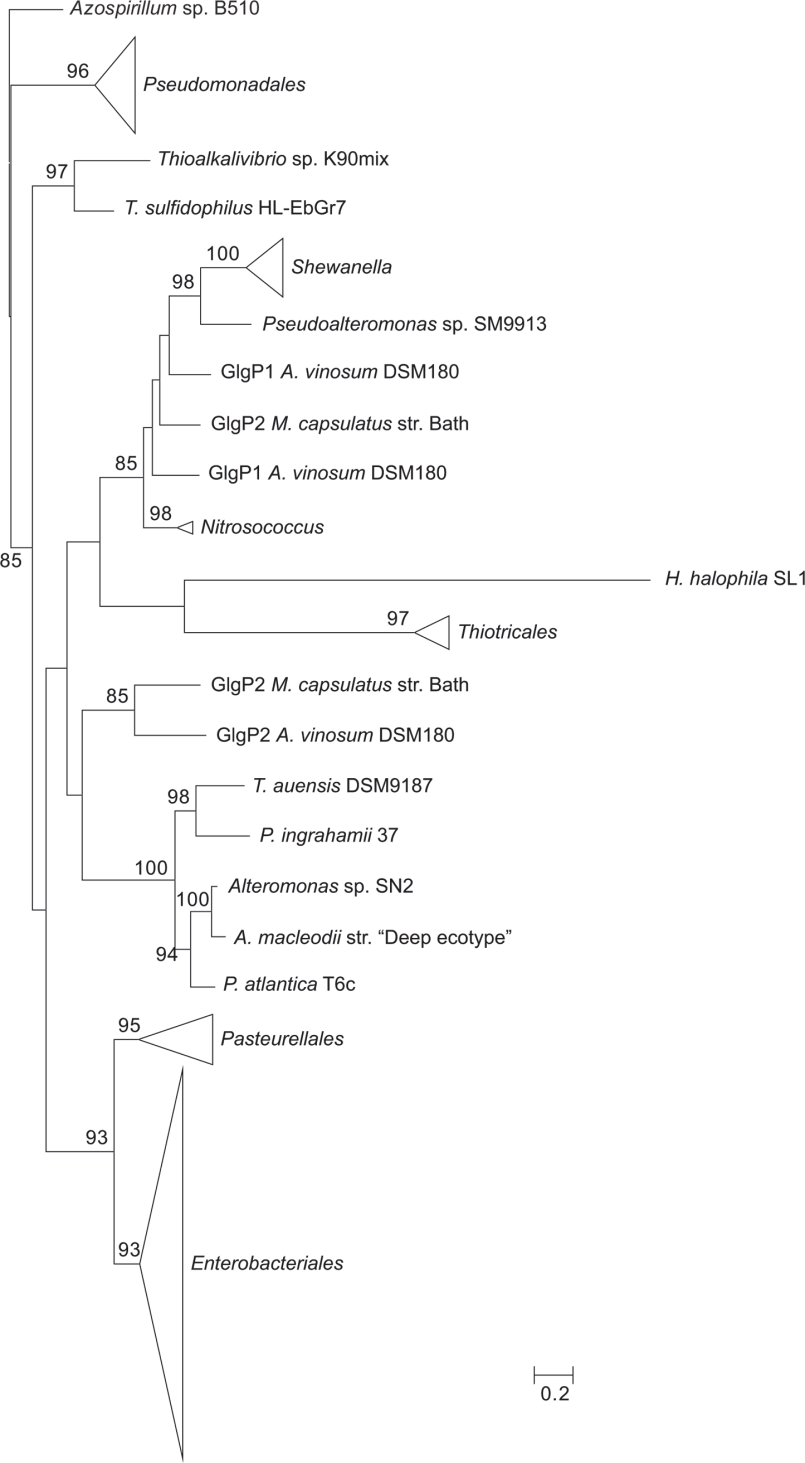
Summarized maximum-likelihood phylogenetic tree of the GlgP amino acid sequences containing all analyzed gammaproteobacterial species. The tree was arbitrarily rooted with the alphaproteobacterial species *Azospirillum* sp. B510. The complete tree is shown in S7 Fig. Bootstrap support values (>70%) are indicated.

The phylogenetic reconstructions were performed with PhyML using the LG+G+I+F model (**Figs. 3–7**). From the inspection of the individual Glg phylogenetic trees some conclusions arose relative to the grouping pattern of the order *Enterobacteriales*. In agreement with the reference 16S rRNA phylogenetic tree of **Fig. 2**, all *Enterobacteriales* Glg sequences clustered with *Pasteurellales* sequences forming monophyletic *Enterobacteriales/Pasteurellales* (E/P) groups in the five Glg phylogenetic trees (**Figs. 3–7; S3–S7 Figs.**). To test whether the topologies of the trees of Glg sequences of the E/P group were compatible with the order of organismal descent inferred from the 16S rRNA phylogenetic analysis, the Shimodaira-Hasegawa test was used. This analysis showed that the topologies of the trees of the five Glg proteins were compatible with the topology of the 16S rRNA phylogenetic tree (p = 0.525, p = 0.462, p = 0.574, p = 0.492 and p = 0.696, for GlgB, GlgX, GlgC, GlgA and GlgP, respectively).

As shown in the 16S rRNA reference tree presented in **Fig. 2**, the E/P group is clustered in a highly supported group (bootstrap support (BS) = 99%) with the orders *Vibrionales, Aeromononadales* and *Alteromonadales* forming the *Vibrionales/Pasteurellales/Enterobacteriales/Aeromonodales/Alteromonadales* (VPEAA) clade. It is worth noting that the monophyly of this VPEAA clade within the *Gammaproteobacteria* has been described in two independent works analyzing the phylogeny of this class [25,29]. However, similar VPEAA clades are not observed in Glg phylogenetic trees since in all cases the E/P group fails to cluster with *Vibrionales, Aeromonadales* and *Alteromonadales* (**Figs. 3–7; S3–S7 Figs.**). Thus, with the exception of the E/P group, lack of congruence is observed between the evolution of *glg* genes and the evolution of the rest of gammaproteobacterial lineages.

### Phylogenetic relationship of E/P Glg proteins with their counterparts outside the *Gammaproteobacteria*

**Figure 8:**
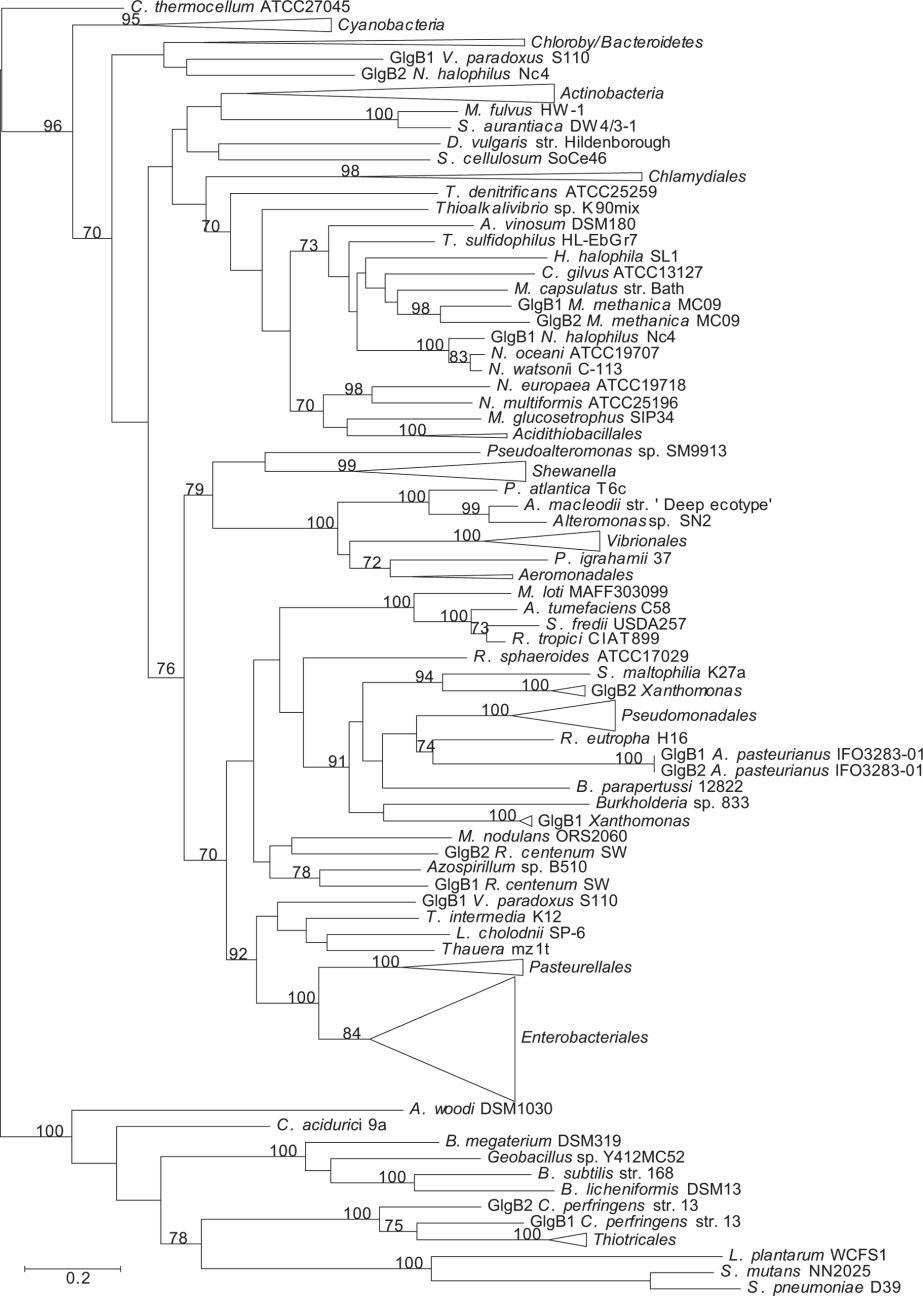
Summarized maximum-likelihood phylogenetic tree of the GlgB amino acid sequences of the analyzed gammaproteobacterial species and of the selected species belonging to main bacterial groups. The tree was midpoint rooted. The complete tree is shown in S9 Fig. Bootstrap support values (>70%) are indicated.

**Figure 9:**
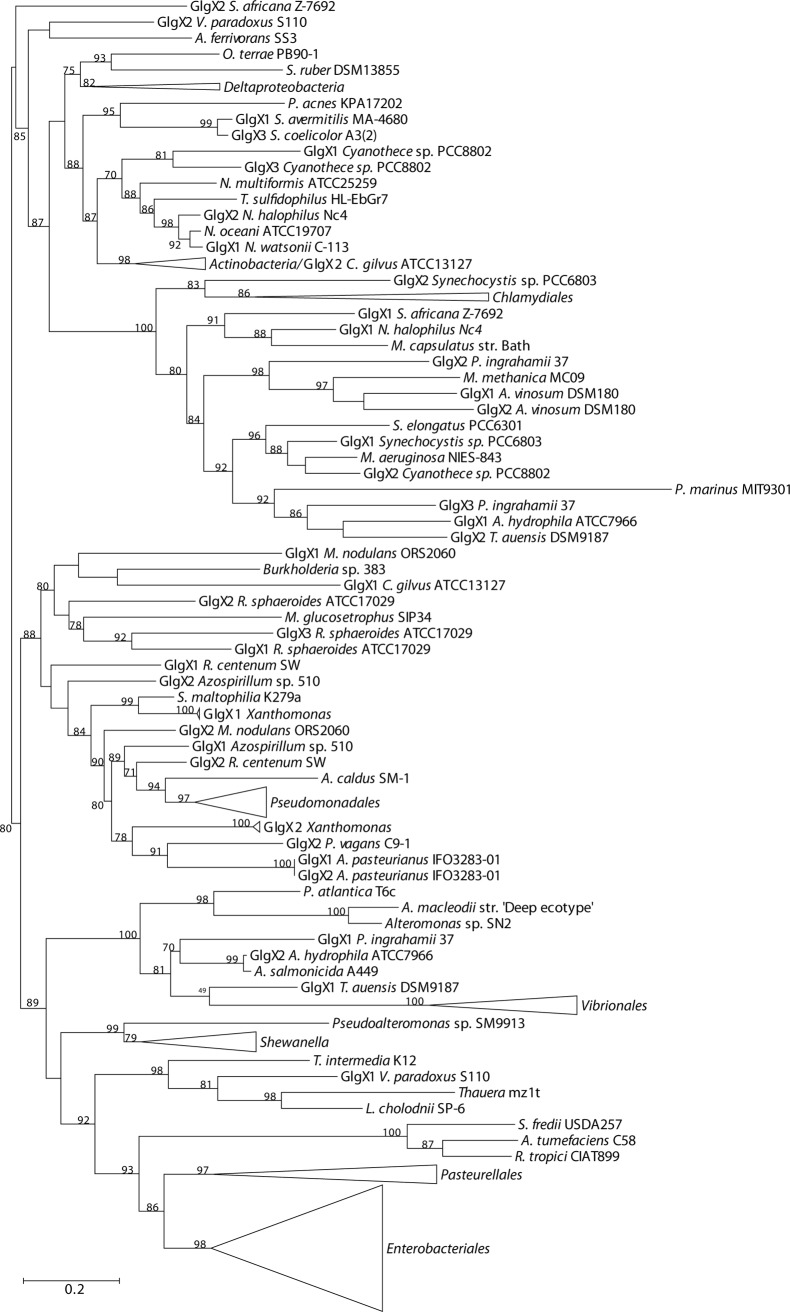
Summarized maximum-likelihood phylogenetic tree of the GlgX amino acid sequences of the analyzed gammaproteobacterial species and of the selected species belonging to main bacterial groups. The tree was midpoint rooted. The complete tree is shown in S10 Fig. Bootstrap support values (>70%) are indicated.

**Figure 10:**
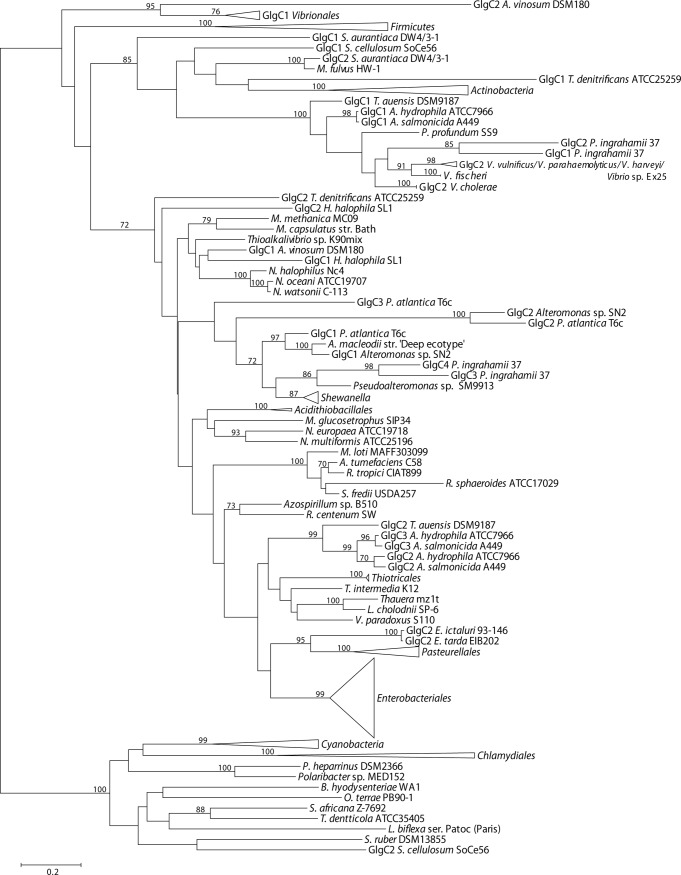
Summarized maximum-likelihood phylogenetic tree of the GlgC amino acid sequences of the analyzed gammaproteobacterial species and of the selected species belonging to main bacterial groups. The tree was midpoint rooted. The complete tree is shown in S11 Fig. Bootstrap support values (>70%) are indicated.

**Figure 11:**
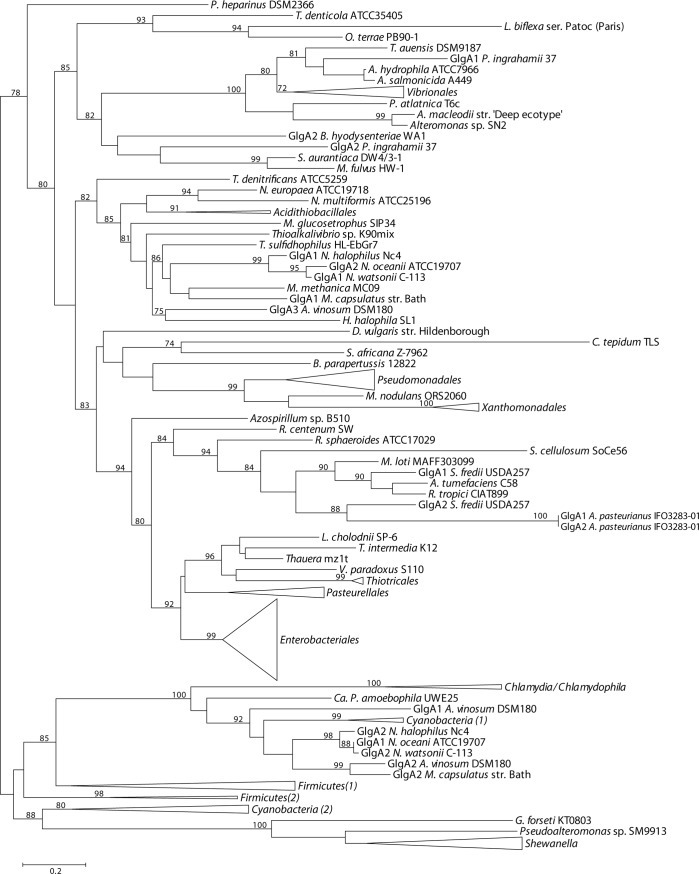
Summarized maximum-likelihood phylogenetic tree of the GlgA amino acid sequences of the analyzed gammaproteobacterial species and of the selected species belonging to main bacterial groups. The tree was midpoint rooted. The complete tree is shown in S12 Fig. Bootstrap support values (>70%) are indicated. The branch of GlgA1 and GlgA2 *A. pasteurianus* IFO3283-01 has been shortened to facilitate its visualization.

**Figure 12:**
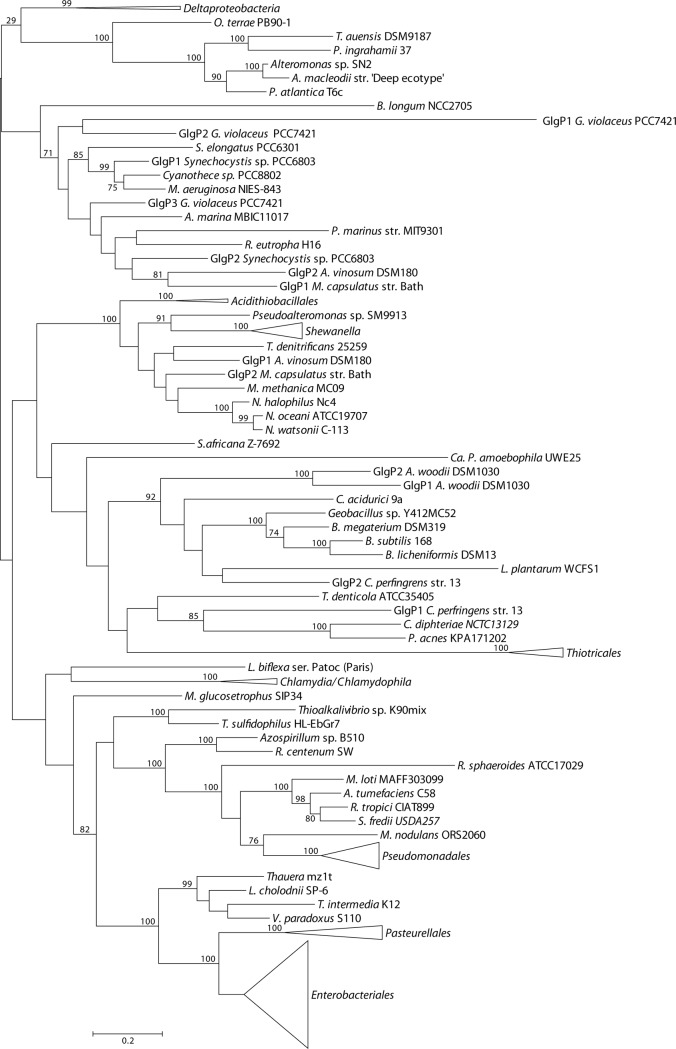
Summarized maximum-likelihood phylogenetic tree of the GlgP amino acid sequences of the analyzed gammaproteobacterial species and of the selected species belonging to main bacterial groups. The tree was midpoint rooted. The complete tree is shown in S13 Fig. Bootstrap support values (>70%) are indicated. The branch of GlgP1 *G. violaceus* PCC7421 has been shortened to facilitate its visualization.

**Figure 13:**
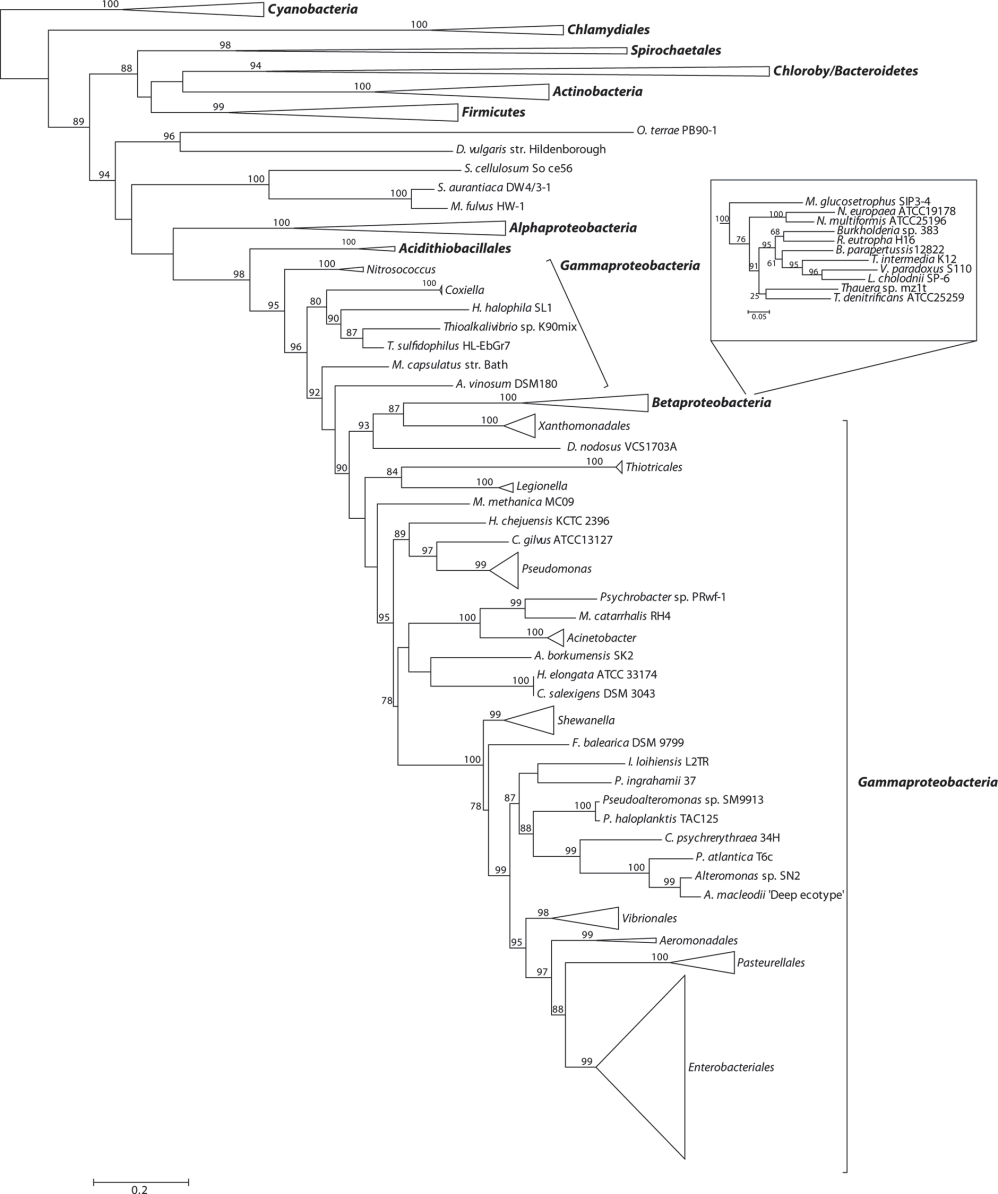
Summarized maximum-likelihood phylogenetic tree for 16S rRNA of the analyzed gammaproteobacterial species and of the selected species belonging to main bacterial groups. Support values for the bootstrap analysis by maximum likelihood are given. The *Gammaproteobacteria* and main bacterial groups are denoted by brackets. The tree was midpoint rooted. The complete tree is shown in S14 Fig.

We performed a phylogenetic reconstruction including both gammaproteobacterial Glg protein sequences and those of 75 species representing the main bacterial groups outside the *Gammaproteobacteria* (**S2 Table**). The Glg sequences thus obtained were aligned with ClustalW and subsequently refined with Gblocks. The resulting data sets consisted of 279 sequences with 406 conserved positions for the GlgB alignment, 257 sequences and 291 conserved positions for the GlgX alignment, 245 sequences and 226 conserved positions for the GlgC alignment, 259 sequences and 157 conserved positions for the GlgA alignment and 217 sequences and 520 conserved positions for the GlgP alignment. The best-fit model for these data sets was LG+G+I+F. The likelihood mapping (using again WAG+G+F) showed a marked decrease in phylogenetic signal compared to the gammaproteobacterial data sets (**S8 Fig.**). The phylogenetic reconstructions were performed as described for the gammaproteobacterial data sets (**Figs. 8–12; S9–S13 Figs.**). Moreover, a new 16S rRNA reference tree including both gammaproteobacterial and representative species of the main bacterial groups outside the *Gammaproteobacteria* was constructed (**Fig. 13; S14 Fig.**).

The above phylogenetic analyses further confirmed the E/P monophyly for the five *glg* genes and the lack of congruence between the Glg phylogenetic trees and the 16S rRNA reference tree (**Figs. 8–13; S9–S14 Figs.**). Remarkably, these analyses also revealed that the Glg proteins of the betaproteobacterial species *Variovorax paradoxus* S110, *Thauera sp.* mz1t, *Leptothrix cholodnii* SP-6 and *Thiomonas intermedia* K12 all clustered with their corresponding homologs of the E/P group, forming a sister clade of E/P in GlgB, GlgA and GlgP phylogenetic trees (**Figs. 8, 11, 12; S9, S12, S13 Figs.**). This close relationship among proteins of such distant lineages could be explained by horizontal gene transfer (HGT). HGT events can be detected by nucleotide compositional analyses. Therefore, compositional similarity analyses of *V. paradoxus* S110, *Thauera sp.* mz1t, *L. cholodnii* SP-6 and *T. intermedia* K12 genomes were carried out using GOHTAM web tool [30]. These analyses showed that *glg* genes of these betaproteobacterial species did not exhibit atypical compositional features in the context of their respective genomes **(S3 Table)**. Finally, it is worth mentioning that among these betaproteobacterial species only *V. paradoxus* S110 possesses additional copies of *glg* genes (*glgB2* and *glgX2*) (see below), which are related with *glgB* and *glgX* of other betaproteobacterial lineages (**Figs. 8, 9; S9, S10 Figs.**).

### Genomic arrangement of *glg* genes

**Figure 14:**
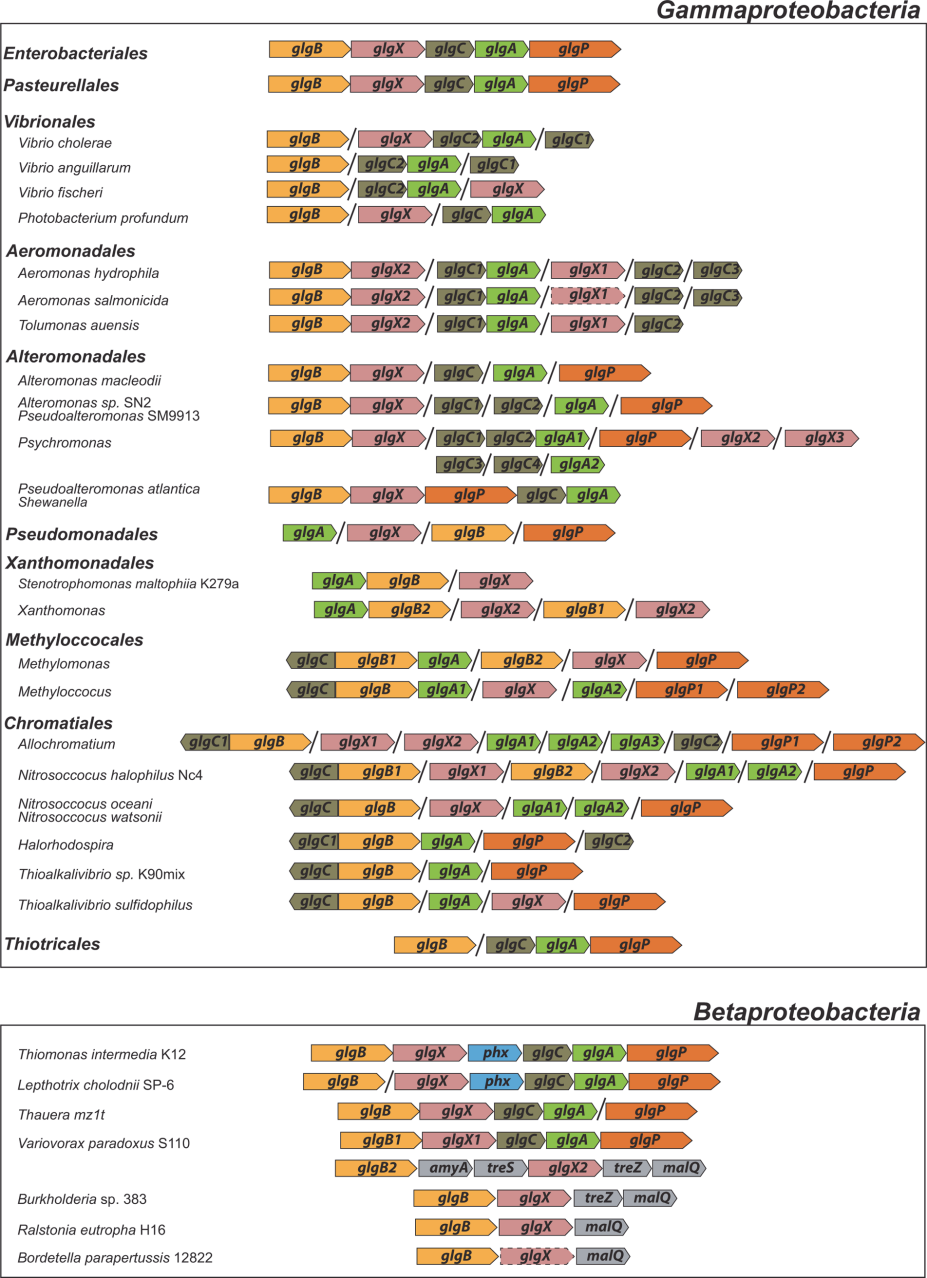
Arrangements of *glg* genes in the specified species. Colors indicate homologous genes. Arrows indicate the direction of transcription. Black lines indicate a physical separation between the genes and dashed lines indicate pseudogenes.

The analysis of the *glg* genes genomic context showed that the *glgBXCAP* arrangement exists not only in *E. coli* but also in all E/P species bearing *glg* genes (**Fig. 14**). This is not surprising since previous studies have shown that the gene order in operon structures in the *Enterobacteriales* and the *Pasteurellales* is well conserved [31,32]. Thus, 56% of operons of *E. coli* (order *Enterobacteriales*) were found to be identical to those of *H. influenzae* (order *Pasteurellales*) [31]. Remarkably, the phylogenetically distant betaproteobacterial species *V. paradoxus* S110, *Thauera sp.* mz1t, *L. cholodnii* SP-6 and *T. intermedia* K12 present a genomic arrangement of *glg* genes very similar to that of E/P (**Fig. 14**). In fact, the *glg* genes in *V. paradoxus S110* are arranged following the *glgBXCAP* order (**Fig. 14**). No other analyzed species of any bacterial group exhibits the *glgBXCAP* arrangement.

As noted above, *V. paradoxus* S110 possesses additional *glgB* and *glgX* copies to those of the *glgBXCAP* cluster (see above). These copies are arranged in a cluster similar to that occurring in the phylogenetically related betaproteobacterial species *Burkholderia sp.* 383, *Ralstonia eutropha* H16 and *Bordetella parapertussis* 12822 (**Figs. 13, 14**). Remarkably, *T. intermedia* K12 and *L. cholodnii* SP-6 present a phosphohexomutase encoding *phx* gene between *glgX* and *glgC* (**Fig. 14**).

Our studies of *glg* arrangements also revealed that *glg* genes are clustered in *glgBXPCA* disposition in the genus *Shewanella* and in the species *P. atlantica* T6c of the order *Alteromonadales* (**Fig. 14**). In addition, species of the orders *Aeromonadales* and *Alteromonadales* share the *glgBX* disposition of *glg* genes (**Fig. 14**), whereas the tandem *glgCA* is conserved in the species belonging to the orders *Vibrionales* and *Aeromonadales* (**Fig. 14**) and *glgCAP* is present in species of *Thiotricales*.

## Discussion

The aim of this work was to elucidate the origin and evolutionary history of *E. coli glg* genes. Our analyses indicate that *glg* genes are present in most gammaproteobacterial lineages, from the earliest to the most recently emerged branches (**Fig. 2**). This widespread distribution in species displaying a wide range of lifestyles suggests that function(s) linked to *glg* genes would provide some advantages for survival under varied and varying environmental conditions. Yet, our analyses have also shown large variability in content, copy number and arrangement of *glg* genes among the different gammaproteobacterial lineages (**S1 Table; Fig. 14**). The phylogenetic analyses carried out in both the *Gammaproteobacteria* and other main bacterial groups revealed an important discordance between the evolution of *glg* genes (**Figs. 3–12; S3–S13 Figs.**) and the evolution of the analyzed bacterial species (**Figs. 2, 13**), thus strongly suggesting that HGT has played an important role in the evolutionary history of *glg* genes. However, a remarkable exception to this rule is constituted by species of the E/P group in which each individual *glg* gene forms a monophyletic group in the corresponding phylogenetic trees that agrees with the expected order of organismal descent as inferred from 16S rRNA phylogenetic analysis (**Figs. 2, 13; S1, S14 Figs.**). Our data strongly suggest that (a) E/P *glg* genes share a common origin in the last common ancestor (LCA) of the E/P group, (b) vertical inheritance has been the primary mode of transmission of these genes throughout evolution in this group and (c) the origin of the *glgBXCAP* cluster could be traced to the LCA of the E/P group as inferred from the same arrangement found in all analyzed E/P species. The complex evolutionary history of *glg* genes in the other bacterial lineages prevented us from further tracing the evolutionary history of gammaproteobacterial *glg* genes.

The most likely evolutionary explanation for the occurrence of species or groups totally or partially lacking *glg* genes is that both complete and partial losses of *glg* genes have occurred independently during the evolution of different gammaproteobacterial lineages. In the analyzed data set, partial *glg* losses are less frequent than the loss of all *glg* genes. In fact, the observed distribution of partial *glg* losses in the different gammaproteobacterial lineages can be explained by seven independent evolutionary events, whereas eighteen independent losses are necessary to explain the distribution of the species lacking all five *glg* genes (indicated by asterisks in **Fig. 2**). Examples of recent losses of all *glg* genes are provided by *H. influenzae* (order *Pasteurellales*) in which one isolate (F3031) lacks all *glg* genes whereas complete sets are present in phylogenetically close isolates such 86-028NP and PittGG (**S1 Table; Fig. 2**). Recent independent losses of all *glg* genes have also occurred in *S. denitrificans* OS217, *S. halifaxensis* HAW-EB4, *S. paeleana* ANG-SO1, *S. sedimis* HAW-EB3, *S. woodyi* ATCC51908, *P. haloplanktis* TAC125 (order *Alteromonadales*), *S. maltophilia* R551-3 (order *Xanthomonadales*) and *Psychrobacter sp. PRwf-1* (order *Pseudomonadales*) (**S1 Table; Fig. 2**). The loss of all *glg* genes is also observed in the marine species belonging to the order *Oceanospirillales*, in the plant pathogenic species of *Xylella*, in the *Acinetobacter* species (which live in the soil and causes infections in humans), in the pathogenic species belonging to the order *Legionellales*, in *P. mirabilis* HI4320 or in the insect endosymbionts of the genera *Buchnera, Blochmannia, Baumannia, Sodalis, Wigglesworthia* and *Photorhabdus* (all belonging to the order *Enterobacteriales*) (**S1 Table; Fig. 2**).

Using 55 complete bacterial genomes (including 11 gammaproteobacterial genomes) Henrissat et al. (2002) [33] reported that bacteria lacking glycogen metabolism are parasitic, symbiotic or fastidious (i. e., difficult to cultivate under laboratory conditions). These authors thus hypothesized that (a) these bacteria rely on their host´s glycogen (or starch) metabolism and (b) the lack of glycogen metabolism is a trait associated with bacterial parasitic or symbiotic behaviors [33]. However, the analysis of a more comprehensive genome set conducted here fails to provide support to this hypothesis at least in the *Gammaproteobacteria*, since the lack of all *glg* genes is observed in species with drastically different lifestyles which are not necessarily parasitic, symbiotic or fastidious such as *P. mirabilis* HI4320, *P. haloplanktis TAC125, S. denitrificans* OS217, *Psychrobacter* sp. PRwf-1 or *A. borkumensis* SK2.

The absence of GlgC and GlgP from some gammaproteobacterial species (**S1 Table**) would imply the loss of ADPG synthesis and glycogen degradation, respectively. Alternatively, it is possible that the absence of these proteins could be compensated by others that are capable of carrying out the same function(s). In this respect we must emphasize that several reports have shown that multiple bacteria possess still unidentified sources of ADPG, other than GlgC, linked to glycogen biosynthesis [5,14,19,20,34]. In the absence of GlgP, it is likely that maltodextrin phosphorylase (MalP), an α-glucan phosphorylase with high similarity to GlgP, could control glycogen degradation [35–37]. On the other hand, the absence of GlgP in some gammaproteobacterial species could indicate that *glg* genes are not related to glycogen metabolism in these species but to other functions in which glycogen degrading enzymes are not necessary, such as the synthesis of exopolysaccharides. In this respect, it must be noted that *Aeromonas hydrophila* (a species lacking GlgP), possesses a GlgA enzyme recognizing both ADPG and UDP-glucose (UDPG) as substrates, and one GlgC homolog displaying UDPG pyrophosphorylase activity rather than ADPG pyrophosphorylase activity involved in the synthesis of a α-branched glucan surface polysaccharide linked to biofilm production [38].

Clustering of genes involved in the same metabolic pathway into a transcriptional unit or operon is frequently observed in bacteria [21–23]. Two models are generally considered to explain operon formation: (i) the selfish operon model postulates that HGT plays a key role in operon evolution [39]; (ii) the co-regulation model proposes that formation of operons facilitates co-regulation [40]. Grouping related genes under a common control mechanism would favour a more efficient regulation of gene expression levels and would allow bacteria to rapidly adapt their metabolism to environmental changes and biochemical needs [41–45]. In the case of *glg* genes, besides the *glgBXCAP* operon present in the gammaproteobacterial E/P group, different *glg* arrangements have been described in other phylogenetically distant bacteria such as in the alphaproteobacterial species *Agrobacterium tumefaciens, Rizhobium tropici* and *Mesorhizobium loti*, and also in the Gram-positive species *Bacillus subtilis* and *Bacillus stearothermophilus* [7]. In this work we have described the presence of the *glgBXPCA* arrangement in species of the gammaproteobacterial genus *Shewanella* and in the species *P. atlantica* T6c (**Fig. 14**). It is worth noting that our phylogenetic analyses showed that *P. atlantica* T6c and *Shewanella glg* genes are not closely related (**Figs. 3–12; S3–S7, S9–S13 Figs.**), which suggests an independent assembly of the *glgBXPCA* cluster in them. Moreover, the *glgBX* arrangement is shared by species of the orders *Aeromonadales* and *Alteromonadales* (**Fig. 14**), the tandem *glgCA* is present in *Vibrionales* and *Aeromonadales* (**Fig. 14**), and the *glgCAP* arrangement is present in *Thiotricales*. Therefore, it seems that along evolution *glg* genes have been clustered together in several independent occasions and in different order, probably responding to different metabolic needs imposed by the environment and lifestyles. Needless to say, further research on the glycogen metabolism and evolution of glycogen operons in a wide range of bacterial species is necessary to confirm (or refute) this hypothesis.

Our study has allowed tracing the evolutionary origin of the *glgBXCAP* operon to the LCA of the E/P group. We can just speculate about the advantages that would account for the widespread preservation of the *glgBXCAP* arrangement throughout the evolution of the E/P group. The transcription of the *glgBXCAP* operon implies the combined expression of glycogen synthetic and degradative enzymes, allowing the simultaneous synthesis and degradation of glycogen. It was previously proposed that the balance of parallel synthesis and degradation pathways serves to maintain glycogen structure and to function as a carbon capacitor thus allowing a sensitive regulation of downstream carbon and energy fluxes [17,46,47]. The resulting turnover of glycogen would entail advantages such as dissipation of excess energy, sensitive regulation and rapid channeling of metabolic intermediates toward various metabolic pathways in response to biochemical needs [48,49]. The latter is particularly relevant, especially when considering that previous reports have shown that glycogen metabolism is highly interconnected with a wide variety of cellular processes [19,50,51].

The observed clustering of the E/P group with phylogenetically distant betaproteobacterial species *V. paradoxus* S110, *L. cholodnii* SP-6, *T. intermedia* K12 and *Thauera sp.* mz1t in the Glg phylogenetic trees (**Figs. 8–12; S9–S13 Figs.**), along with the presence of *glgBXCAP* and/or gene clusters similar to *glgBXCAP* exclusively in these betaproteobacterial species (**Fig. 14**) can be explained by (i) an ancestral duplication of the *glgBXCAP* cluster in their LCA and subsequent loss in all intervening lineages, (ii) two independent HGT events of the *glgBXCAP* cluster from E/P, the first one to the common ancestor of *V. paradoxus* S110, *L. cholodnii* SP-6 and *T. intermedia* K12, and the second one to *Thauera sp*. mz1t, (iii) an HGT event of the *glgBXCAP* cluster from E/P to the common ancestor of *V. paradoxus* S110, *L. cholodnii* SP-6 and *T. intermedia* K12 or to *Thauera sp.* mz1t, and a second HGT event between them, or (iv) an HGT event of the *glgBXCAP* cluster from E/P to the LCA of the four betaproteobacterial species and followed by subsequent multiple, independent losses in the species *Burkholderia sp. 383, R. eutropha* H16, *B. parapertussis* 12822 and *Thiobacillus denitrificans* ATCC25259 (**see Fig. 13**). Previous studies based on parametric methods and aimed at predicting horizontally transferred genes did not identify the *glg* genes as horizontally transferred in the above betaproteobacterial species [52,53], thus favoring the hypothesis of the occurrence of an ancestral duplication of *glg* genes. Consistently, compositional analysis of *V. paradoxus* S110, *L. cholodnii* SP-6, *T. intermedia* K12 and *Thauera sp.* mz1t carried out in this work **(S3 Table)** did not identify *glg* genes as exhibiting atypical compositional features. However, we must emphasize that HGT events produce a localized nucleotide bias that tends to disappear in a relatively short period of evolutionary time, a process commonly known as amelioration [54]. Thus, failure of parametric methods in identifying *glg* genes as horizontally transferred in the above four betaproteobacterial species can be explained by this phenomenon in a situation where the putative HGT event(s) would have occurred before the differentiation of these betaproteobacterial lineages. Although data available cannot unequivocally confirm or rule out any of the above scenarios, it must be noted that the second and the third scenarios (both encompassing HGT events) are the most parsimonious since they would imply only two independent evolutionary events whereas the first would imply multiple and independent losses in other lineages, and the fourth requires five independent events (one HGT and four independent losses).

We must emphasize that *V. paradoxus* S110 possesses additional copies of both *glgB* and *glgX* (**Figs. 8, 9; S9, S10 Figs.**), which are very distant to their own homologs related with the E/P group and located in gene clusters similar to those found in their phylogenetically related betaproteobacterial species *Burkholderia sp.* 383, *R. eutropha* H16, and *B. parapertussis* 12822 (**Fig. 14**). This reinforces the possibility of an horizontal transfer of the complete *glgBXCAP* operon from E/P to the ancestor of *V. paradoxus* S110, *L. cholodnii* SP-6, *T. intermedia* K12 and/or to *Thauera sp.* mz1t. Subsequent gene rearrangements led to the clusters observed in these species. These rearrangements include the relocation of *glgB* in *L. cholodnii* SP-6 and of *glgP* in *Thauera* mz1t, as well as the acquisition of *phx* genes in *T. intermedia* K12 and *L. cholodnii* SP-6.

In agreement with researchers favoring a view in which HGT has played a major continuous role in evolution [55–59] our results indicate its prevalence in the evolution and distribution of *glg* genes within Bacteria. Two main pieces of evidence support this conclusion. First, the phylogenetic analysis of the five Glg proteins shows that the evolution of *glg* genes is in clear conflict with the order of organismal descent, inferred from the 16S rRNA analysis. Second, in species possessing more than one copy of a given *glg* gene, some of the copies are related to homologous copies of phylogenetically distant species as for example *glgX2* of the gammaproteobacterial species *Pantoea vagans* C9-1 (order *Enterobacteriales*) which is more related to its homolog present in the alphaproteobacterial species *Acetobacter pasteurianus* IFO 3283-01 (**Fig. 11; S8 Fig.**), *glgX2* of *Cellvibrio gilvus* ATCC13127 (gammaproteobacterial order *Pseudomonadales*) which is more related with homologs from *Actinobacteria* (**Fig. 11; S8 Fig.**), and *glgC2* of *Edwardsiella* (gammaproteobacterial order *Enterobacteriales*) which is more related with homologs of the *Pasteurellales* (**Fig. 12; S9 Fig.**). Moreover, our results also suggest the occurrence of an horizontal transfer of the complete *glgBXCAP* operon from E/P group to some *Betaproteobacteria*, indicating that HGT is not restricted to individual *glg* genes, adding more complexity to the evolution of these genes.

## Materials and Methods

### Sequences

We have analyzed the complete genomes of 265 different gammaproteobacterial species and strains (see **S1 Table**) and of 75 species of the main bacterial groups outside the *Gammaproteobacteria* (see **S2 Table**) retrieved from the NCBI repository. GlgB, GlgX, GlgC, GlgA, and GlgP encoding genes were retrieved from whole genomes by using PSI-BLAST [60]. The PSI-BLAST searches were performed against the GenBank database using the *E. coli* GlgB, GlgX, GlgC, GlgA and GlgP (accession numbers NP_417890.1, NP_417889.1, NP_417888.1, NP_417887.1 and NP_417886.1 respectively) as query sequences with default settings. From all the sequences retrieved, only those from whole genomes were selected, and these were subsequently filtered attending to a coverage of at least 75% and an identity of at least 35% of the query sequence. The corresponding 16S rRNA sequences from each genome were downloaded using the tools implemented in the Ribosomal Database Project II (RDB-II) [61].

### Alignments and phylogenetic information analyses

Multiple alignments were obtained with ClustalW [62] and were manually corrected. Positions of uncertain homology and extensive gaps were removed using GBLOCKS with default settings [63]. The final multiple alignments used for the analyses are available from the authors upon request. The best-fit models of amino acid substitution were determined using the program ProtTest [64]. The Akaike Information Criterion (AIC), which allows for a comparison of likelihoods from non-tested models, was adopted to select the best model [65]. For the five proteins the model LG [66] with a discrete gamma distribution to account for heterogeneity in evolutionary rates among sites, an estimation of the proportion of invariant sites and the empirical frequencies of amino acids (LG+G+I+F) was identified as the best-fit model. The phylogenetic signal contained in the different data sets was assessed by likelihood-mapping [67] using TreePuzzle 5.2 [68]. This method evaluates the resolution in quartets generated from combinations of the different sequences under study. Values below 90% resolved quartets were considered to indicate a low phylogenetic signal. In this evaluation we used the WAG model [28] of amino acid evolution and a discrete gamma distribution to account for heterogeneity in evolutionary rates among positions in the multiple alignments (**S2, S8 Figs.**).

### Phylogenetic reconstructions

The model selected by ProtTest was implemented in PhyML 3.0 [69] to obtain maximum likelihood (ML) trees for the different multiple alignments. Bootstrap support values were obtained from 1000 pseudorandom replicates.

To obtain the phylogenetic reference trees encompassing all the analyzed species, sequences of genes encoding 16S rRNA were obtained using the tools implemented in the RDB-II. The sequences were aligned using the aligner implemented in RDB-II. The best fit-model of nucleotide substitution was selected using jModelTest [70] with the AIC criterion. The phylogenetic relationships were inferred using PhyML with the GTR model of nucleotide substitution and the proportion of invariant and rate heterogeneity categories estimated from the data set. Bootstrap support values were obtained from 1000 pseudorandom replicates.

For the E/P group we carried out a comparison between each *glg* gene tree and the 16S rRNA (reference tree) topology. The Shimodaira-Hasegawa´s test (SH test) [71] as implemented in the program TreePuzzle 5.2 was used to determine whether the likelihood of the data associated to each tree was significantly different at an alpha level of 0.05 (a value above the threshold indicating a non-significant difference). Phylogenetic trees of 16S rRNA sequences were obtained with the Tree Builder tool of the RDB-II and compared to ML phylogenetic trees of the corresponding Glg sequences from E/P species.

### Compositional analyses

Nucleotide compositional analyses were carried out using the GOHTAM web tool [30]. This web tool allows to identify genomic regions and/or genes that exhibit atypical features compared to the rest of the sequence. The detection is based on a combination of the genomic signature [72,73] and a codon usage method [74].

## Supporting Information

S1 Table Summary of gammaproteobacterial species studied in the present work, their assigned order, and number of copies of each *glg* gene in their genomes. In the case of endosymbionts of insects, the corresponding host species is also indicated between brackets.
(DOCX)

S2 Table Summary of the non-*Gammaproteobacteria* species studied in the present work and the number of copies of the different *glg* genes in their genomes. (DOCX)

S3 Table Regions and/or genes of the betaproteobacterial species *V. paradoxus* S110, *T. intermedia* K12, *L. cholodnii* SP-6 and *Thauera* mz1t that exhibit atypical compositional features compared to the their respective genome. (XLS)

S1 Fig Maximum likelihood phylogenetic tree for 16S rRNA of the analyzed gammaproteobacterial species. Support values >70% for the bootstrap analysis by maximum likelihood are given. The tree was rooted with the alphaproteobacterial species *Azospirillum sp. B510*.
(EPS)

S2 Fig Likelihood mapping analysis of gammaproteobacterial data set. The regions at the corners of the triangles correspond to the three possible tree topologies for a quartet; the lateral regions to partly resolved trees and the central region to unresolved trees. The numbers indicate the percentage of quartets falling in each region.
(EPS)

S3 Fig Maximum likelihood phylogenetic tree for gammaproteobacterial GlgB sequences used in this study. The tree was rooted with the alphaproteobacterial species *Azospirillum* sp. B510. Support values >70% for the bootstrap analysis by maximum likelihood are given.
(EPS)

S4 Fig Maximum likelihood phylogenetic tree for gammaproteobacterial GlgX sequences used in this study. The tree was rooted with the alphaproteobacterial species *Azospirillum* sp. B510. Support values >70% for the bootstrap analysis by maximum likelihood are given.
(EPS)

S5 Fig Maximum likelihood phylogenetic tree for gammaproteobacterial GlgC sequences used in this study. The tree was rooted with the alphaproteobacterial species *Azospirillum* sp. B510. Support values >70% for the bootstrap analysis by maximum likelihood are given.
(EPS)

S6 Fig Maximum likelihood phylogenetic tree for gammaproteobacterial GlgA sequences used in this study. The tree was rooted with the alphaproteobacterial species *Azospirillum* sp. B510. Support values >70% for the bootstrap analysis by maximum likelihood are given.
(EPS)

S7 Fig Maximum likelihood phylogenetic tree for gammaproteobacterial GlgP sequences used in this study. The tree was rooted with the alphaproteobacterial species *Azospirillum* sp. B510. Support values >70% for the bootstrap analysis by maximum likelihood are given.
(EPS)

S8 Fig Likelihood mapping analysis of the data set including the analyzed gammaproteobacterial species and representative species of the main bacterial groups. The regions at the corners of the triangles correspond to the three possible tree topologies for a quartet; the lateral regions to partly resolved trees and the central region to unresolved trees. The numbers indicate the percentage of quartets falling in each region.
(EPS)

S9 Fig Maximum likelihood phylogenetic tree for GlgB sequences of the analyzed gammaproteobacterial species and representative species of the main bacterial groups. The tree is midpoint rooted. Support values >70% for the bootstrap analysis by maximum likelihood are given.
(EPS)

S10 Fig Maximum likelihood phylogenetic tree for GlgX sequences of the analyzed gammaproteobacterial species and representative species of the main bacterial groups. The tree is midpoint rooted. Support values >70% for the bootstrap analysis by maximum likelihood are given.
(EPS)

S11 Fig Maximum likelihood phylogenetic tree for GlgC sequences of the analyzed gammaproteobacterial species and representative species of the main bacterial groups. The tree is midpoint rooted. Support values >70% for the bootstrap analysis by maximum likelihood are given.
(EPS)

S12 Fig Maximum likelihood phylogenetic tree for GlgA sequences of the analyzed gammaproteobacterial species and representative species of the main bacterial groups. The tree is midpoint rooted. Support values >70% for the bootstrap analysis by maximum likelihood are given.
(EPS)

S13 Fig Maximum likelihood phylogenetic tree for GlgP sequences of the analyzed gammaproteobacterial species and representative species of the main bacterial groups. The tree is midpoint rooted. Support values >70% for the bootstrap analysis by maximum likelihood are given.
(EPS)

S14 Fig Maximum likelihood phylogenetic tree for 16S rRNA of the analyzed gammaproteobacterial species and of the selected species belonging to main bacterial groups. Support values >70% for the bootstrap analysis by maximum likelihood are given. The tree was midpoint rooted.
(EPS)
