# Supplementary material for: Comparative Genomic and Phylogenetic Analyses of Gammaproteobacterial glg Genes Traced the Origin of the Escherichia coli Glycogen glgBXCAP Operon to the Last Common Ancestor of the Sister Orders Enterobacteriales and Pasteurellales
Source: PLoS One. 2015 Jan 21;10(1):e0115516. doi: 10.1371/journal.pone.0115516 (PMC4301808; doi:10.1371/journal.pone.0115516)
Supplement: S1 Table — In the case of endosymbionts of insects, the corresponding host species is also indicated between brackets. (DOCX) [file pone.0115516.s001.docx]

**Table S1**

| **Order** | **Species** | **Genome**  **accesion number** | **Copy number** | | | | |
| --- | --- | --- | --- | --- | --- | --- | --- |
|  |  |  | ***glgB*** | ***glgX*** | ***glgC*** | ***glgA*** | ***glgP*** |
| *Enterobacteriales* | *Baumannia cicadellinicola* str. Hc *(Homalodisca coagulata)* | NC_007984.1 | 0 | 0 | 0 | 0 | 0 |
|  | *Buchnera aphidicola (Cinara tujafilina)* | NC_015662.1 | 0 | 0 | 0 | 0 | 0 |
|  | *Buchnera aphidicola* str. 5A *(Acyrthosiphon pisum)* | NC_015662.1 | 0 | 0 | 0 | 0 | 0 |
|  | *Buchnera aphidicola* str. APS *(Acyrthosiphon pisum)* | NC_002528.1 | 0 | 0 | 0 | 0 | 0 |
|  | *Buchnera aphidicola* str. Bp *(Baizongia pistaciae)* | NC_004545.1 | 0 | 0 | 0 | 0 | 0 |
|  | *Buchnera aphidicola* str. Cc *(Cinara cedri)* | NC_008513.1 | 0 | 0 | 0 | 0 | 0 |
|  | *Buchnera aphidicola* str. JF98 *(Acyrthosiphon pisum)* | NC_017254.1 | 0 | 0 | 0 | 0 | 0 |
|  | *Buchnera aphidicola* str. JF99 *(Acyrthosiphon pisum)* | NC_017253.1 | 0 | 0 | 0 | 0 | 0 |
|  | *Buchnera aphidicola* str. LL01 *(Acyrthosiphon pisum)* | NC_017255.1 | 0 | 0 | 0 | 0 | 0 |
|  | *Buchnera aphidicola* str. Sg *(Schizaphis graminum)* | NC_004061.1 | 0 | 0 | 0 | 0 | 0 |
|  | *Buchnera aphidicola* str. TLW03 *(Acyrthosiphon pisum)* | NC_017252.1 | 0 | 0 | 0 | 0 | 0 |
|  | *Buchnera aphidicola* str. Tuc7 *(Acyrthosiphon pisum)* | NC_011834.1 | 0 | 0 | 0 | 0 | 0 |
|  | *Candidatus Blochmannia floridanus (Formica ligniperda)* | NC_005061.1 | 0 | 0 | 0 | 0 | 0 |
|  | *Candidatus Blochmannia pennsylvanicus* str. BPEN *(Camponotus pennsylvanicus)* | NC_007292.1 | 0 | 0 | 0 | 0 | 0 |
|  | *Candidatus Blochmannia vafer* str. BVAF *(Camponotus vafer)* | NC_014909.2 | 0 | 0 | 0 | 0 | 0 |
|  | *Candidatus Hamiltonella defensa* 5AT *(Acyrthosiphon pisum)* | NC_012751.1 | 0 | 0 | 0 | 0 | 0 |
|  | *Citrobacter koseri* ATCC BAA-895 | NC_009792.1 | 1 | 1 | 1 | 1 | 1 |
|  | *Citrobacter rodentium* ICC168 | NC_013716.1 | 1 | 1 | 1 | 1 | 1 |
|  | *Dickeya dadantii* 3937 | NC_014500.1 | 1 | 1 | 1 | 1 | 1 |
|  | *Dickeya dadantii* Ech586 | NC_013592.1 | 1 | 1 | 1 | 1 | 1 |
|  | *Dickeya dadantii* Ech703 | NC_012880.1 | 1 | 1 | 1 | 1 | 1 |
|  | *Dickeya zeae* Ech1591 | NC_012912.1 | 1 | 1 | 1 | 1 | 1 |
|  | *Edwardsiella ictaluri 93-146* | NC_012779.2 | 1 | 1 | 2 | 1 | 1 |
|  | *Edwardsiella tarda EIB202* | NC_013508.1 | 1 | 1 | 2 | 1 | 1 |
|  | *Enterobacter aerogenes KCTC 2190* | NC_015663.1 | 1 | 1 | 1 | 1 | 1 |
|  | *Enterobacter cloacae SCF1* | NC_014618.1 | 1 | 1 | 1 | 1 | 1 |
|  | *Enterobacter cloacae subsp. cloacae ATCC 13047* | NC_014121.1 | 1 | 1 | 1 | 1 | 1 |
|  | *Enterobacter sp. 638* | NC_009436.1 | 1 | 1 | 1 | 1 | 1 |
|  | *Erwinia amylovora ATCC 49946* | NC_013971.1 | 1 | 1 | 1 | 1 | 1 |
|  | *Erwinia amylovora CFBP1430* | NC_013961.1 | 1 | 1 | 1 | 1 | 1 |
|  | *Erwinia billingiae Eb661* | NC_014306.1 | 1 | 1 | 1 | 1 | 1 |
|  | *Erwinia pyrifoliae Ep1/96* | NC_012214.1 | 1 | 1 | 1 | 1 | 1 |
|  | *Erwinia tasmaniensis Et1/99* | NC_010694.1 | 1 | 1 | 1 | 1 | 1 |
|  | *Escherichia coli 'BL21-Gold(DE3)pLysS AG'* | NC_012947.1 | 1 | 1 | 1 | 1 | 1 |
|  | *Escherichia coli 536* | NC_008253.1 | 1 | 1 | 1 | 1 | 1 |
|  | *Escherichia coli 55989* | NC_011748.1 | 1 | 1 | 1 | 1 | 1 |
|  | *Escherichia coli APEC O1* | NC_008563.1 | 1 | 1 | 1 | 1 | 1 |
|  | *Escherichia coli ATCC 8739* | NC_010468.1 | 1 | 1 | 1 | 1 | 1 |
|  | *Escherichia coli B str. REL606* | NC_012967.1 | 1 | 1 | 1 | 1 | 1 |
|  | *Escherichia coli BW2952* | NC_012759.1 | 1 | 1 | 1 | 1 | 1 |
|  | *Escherichia coli CFT073* | NC_004431.1 | 1 | 1 | 1 | 1 | 1 |
|  | *Escherichia coli E24377A* | NC_009801.1 | 1 | 1 | 1 | 1 | 1 |
|  | *Escherichia coli ED1a* | NC_011745.1 | 1 | 1 | 1 | 1 | 1 |
|  | *Escherichia coli HS* | NC_009800.1 | 1 | 1 | 1 | 1 | 1 |
|  | *Escherichia coli IAI1* | NC_011741.1 | 1 | 1 | 1 | 1 | 1 |
|  | *Escherichia coli IAI39* | NC_011750.1 | 1 | 1 | 1 | 1 | 1 |
|  | *Escherichia coli O103:H2 str. 12009* | NC_013353.1 | 1 | 1 | 1 | 1 | 1 |
|  | *Escherichia coli O111:H- str. 11128* | NC_013364.1 | 1 | 1 | 1 | 1 | 1 |
|  | *Escherichia coli O127:H6 str. E2348/69* | NC_011601.1 | 1 | 1 | 1 | 1 | 1 |
|  | *Escherichia coli O157:H7 str. EC4115* | NC_011353.1 | 1 | 1 | 1 | 1 | 1 |
|  | *Escherichia coli O157:H7 str. EDL933* | NC_002655.2 | 1 | 1 | 1 | 1 | 1 |
|  | *Escherichia coli O157:H7 str. Sakai* | NC_002695.1 | 1 | 1 | 1 | 1 | 1 |
|  | *Escherichia coli O157:H7 str. TW14359* | NC_013008.1 | 1 | 1 | 1 | 1 | 1 |
|  | *Escherichia coli O26:H11 str. 11368* | NC_013361.1 | 1 | 1 | 1 | 1 | 1 |
|  | *Escherichia coli O55:H7 str. CB9615* | NC_013941.1 | 1 | 1 | 1 | 1 | 1 |
|  | *Escherichia coli S88* | NC_011742.1 | 1 | 1 | 1 | 1 | 1 |
|  | *Escherichia coli SE11* | NC_011415.1 | 1 | 1 | 1 | 1 | 1 |
|  | *Escherichia coli SMS-3-5* | NC_010498.1 | 1 | 1 | 1 | 1 | 1 |
|  | *Escherichia coli str. K-12 substr. DH10B* | NC_010473.1 | 1 | 1 | 1 | 1 | 1 |
|  | *Escherichia coli str. K-12 substr. MG1655* | NC_000913.3 | 1 | 1 | 1 | 1 | 1 |
|  | *Escherichia coli UMN026* | NC_011751.1 | 1 | 1 | 1 | 1 | 1 |
|  | *Escherichia coli UTI89* | NC_007946.1 | 1 | 1 | 1 | 1 | 1 |
|  | *Escherichia fergusonii ATCC 35469* | NC_011740.1 | 1 | 1 | 1 | 1 | 1 |
|  | *Klebsiella pneumoniae 342* | NC_011283.1 | 1 | 1 | 1 | 1 | 1 |
|  | *Klebsiella pneumoniae subsp. pneumoniae MGH 78578* | NC_009648.1 | 1 | 1 | 1 | 1 | 1 |
|  | *Klebsiella pneumoniae subsp. pneumoniae NTUH-K2044* | NC_012731.1 | 1 | 1 | 1 | 1 | 1 |
|  | *Klebsiella variicola At-22* | NC_013850.1 | 1 | 1 | 1 | 1 | 1 |
|  | *Pantoea ananatis LMG 20103* | NC_013956.2 | 1 | 1 | 1 | 1 | 1 |
|  | *Pantoea sp. At-9b* | NC_014837.1 | 1 | 1 | 1 | 1 | 1 |
|  | *Pantoea vagans C9-1* | NC_014562.1 | 1 | 2 | 1 | 1 | 1 |
|  | *Pectobacterium atrosepticum SCRI1043* | NC_004547.2 | 1 | 1 | 1 | 1 | 1 |
|  | *Pectobacterium carotovorum subsp. carotovorum PC1* | NC_012917.1 | 1 | 1 | 1 | 1 | 1 |
|  | *Pectobacterium wasabiae WPP163* | NC_013421.1 | 1 | 1 | 1 | 1 | 1 |
|  | *Photorhabdus asymbiotica (Steinernema spp.)* | NC_012962.1 | 0 | 0 | 0 | 0 | 0 |
|  | *Photorhabdus luminescens subsp. laumondii TTO1 (Steinernema spp.)* | NC_005126.1 | 0 | 0 | 0 | 0 | 0 |
|  | *Proteus mirabilis HI4320* | NC_010554.1 | 0 | 0 | 0 | 0 | 0 |
|  | *Rahnella sp. Y9602* | NC_015061.1 | 1 | 1 | 1 | 1 | 1 |
|  | *Salmonella bongori NCTC 12419* | NC_015761.1 | 1 | 1 | 1 | 1 | 1 |
|  | *Salmonella enterica subsp. arizonae serovar 62:z4,z23:-- str. RSK2980* | NC_010067.1 | 1 | 1 | 1 | 1 | 1 |
|  | *Salmonella enterica subsp. enterica serovar Agona str. SL483* | NC_011149.1 | 1 | 1 | 1 | 1 | 1 |
|  | *Salmonella enterica subsp. enterica serovar Choleraesuis str. SC-B67* | NC_006905.1 | 1 | 1 | 1 | 1 | 1 |
|  | *Salmonella enterica subsp. enterica serovar Dublin str. CT_02021853* | NC_011205.1 | 1 | 1 | 1 | 1 | 1 |
|  | *Salmonella enterica subsp. enterica serovar Enteritidis str. P125109* | NC_011294.1 | 1 | 1 | 1 | 1 | 1 |
|  | *Salmonella enterica subsp. enterica serovar Gallinarum str. 287/91* | NC_011274.1 | 1 | 1 | 1 | 1 | 1 |
|  | *Salmonella enterica subsp. enterica serovar Heidelberg str. SL476* | NC_011083.1 | 1 | 1 | 1 | 1 | 1 |
|  | *Salmonella enterica subsp. enterica serovar Newport str. SL254* | NC_011080.1 | 1 | 1 | 1 | 1 | 1 |
|  | *Salmonella enterica subsp. enterica serovar Paratyphi A str. AKU_12601* | NC_011147.1 | 1 | 1 | 1 | 1 | 1 |
|  | *Salmonella enterica subsp. enterica serovar Paratyphi A str. ATCC 9150* | NC_006511.1 | 1 | 1 | 1 | 1 | 1 |
|  | *Salmonella enterica subsp. enterica serovar Paratyphi C strain RKS4594* | NC_012125.1 | 1 | 1 | 1 | 1 | 1 |
|  | *Salmonella enterica subsp. enterica serovar Schwarzengrund str. CVM19633* | NC_011094.1 | 1 | 1 | 1 | 1 | 1 |
|  | *Salmonella enterica subsp. enterica serovar Typhi str. CT18* | NC_003198.1 | 1 | 1 | 1 | 1 | 1 |
|  | *Salmonella enterica subsp. enterica serovar Typhi str. Ty2* | NC_004631.1 | 1 | 1 | 1 | 1 | 1 |
|  | *Salmonella enterica subsp. enterica serovar Typhimurium str. LT2* | NC_003197.1 | 1 | 1 | 1 | 1 | 1 |
|  | *Serratia proteamaculans 568* | NC_009832.1 | 1 | 1 | 1 | 1 | 1 |
|  | *Shigella boydii CDC 3083-94* | NC_010658.1 | 1 | 1 | 1 | 1 | 1 |
|  | *Shigella boydii Sb227* | NC_007613.1 | 1 | 1 | 1 | 1 | 1 |
|  | *Shigella dysenteriae Sd197* | NC_007606.1 | 1 | 1 | 1 | 1 | 1 |
|  | *Shigella flexneri 2a str. 2457T* | NC_004741.1 | 1 | 1 | 1 | 1 | 1 |
|  | *Shigella flexneri 5 str. 8401* | NC_008258.1 | 1 | 1 | 1 | 1 | 1 |
|  | *Shigella sonnei Ss046* | NC_007384.1 | 1 | 1 | 1 | 1 | 1 |
|  | *Sodalis glossinidius str. 'morsitans' (Glossina spp.)* | NC_007712.1 | 0 | 0 | 0 | 0 | 0 |
|  | *Wigglesworthia glossinidia (Glossina brevipalpis)* | NC_004344.2 | 0 | 0 | 0 | 0 | 0 |
|  | *Xenorhabdus bovienii SS-2004 (Steinernema spp.)* | NC_013892.1 | 0 | 0 | 0 | 0 | 0 |
|  | *Xenorhabdus nematophila ATCC 19061 (Steinernema spp.)* | NC_014228.1 | 0 | 0 | 0 | 0 | 0 |
|  | *Yersinia enterocolitica subsp. enterocolitica 8081* | NC_008800.1 | 1 | 1 | 1 | 1 | 1 |
|  | *Yersinia enterocolitica subsp. palearctica 105.5R(r)* | NC_015475.1 | 1 | 1 | 1 | 1 | 1 |
|  | *Yersinia pestis Angola* | NC_010159.1 | 1 | 1 | 1 | 1 | 1 |
|  | *Yersinia pestis Antiqua* | NC_008150.1 | 1 | 1 | 1 | 1 | 1 |
|  | *Yersinia pestis biovar Microtus str. 91001* | NC_005810.1 | 1 | 1 | 1 | 1 | 1 |
|  | *Yersinia pestis CO92* | NC_003143.1 | 1 | 1 | 1 | 1 | 1 |
|  | *Yersinia pestis KIM 10* | NC_004088.1 | 1 | 1 | 1 | 1 | 1 |
|  | *Yersinia pestis Nepal516* | NC_008149.1 | 1 | 1 | 1 | 1 | 1 |
|  | *Yersinia pestis Pestoides F* | NC_009381.1 | 1 | 1 | 1 | 1 | 1 |
|  | *Yersinia pestis Z176003* | NC_014029.1 | 1 | 1 | 1 | 1 | 1 |
|  | *Yersinia pseudotuberculosis IP 31758* | NC_009708.1 | 1 | 1 | 1 | 1 | 1 |
|  | *Yersinia pseudotuberculosis IP 32953* | NC_006155.1 | 1 | 1 | 1 | 1 | 1 |
|  | *Yersinia pseudotuberculosis PB1/+* | NC_010634.1 | 1 | 1 | 1 | 1 | 1 |
|  | *Yersinia pseudotuberculosis YPIII* | NC_010465.1 | 1 | 1 | 1 | 1 | 1 |
| *Pasteurellales* | *Actinobacillus pleuropneumoniae serovar 3 str. JL03* | NC_010278.1 | 1 | 1 | 1 | 1 | 1 |
|  | *Actinobacillus pleuropneumoniae serovar 5b str. L20* | NC_009053.1 | 1 | 1 | 1 | 1 | 1 |
|  | *Actinobacillus pleuropneumoniae serovar 7 str. AP76* | NC_010939.1 | 1 | 1 | 1 | 1 | 1 |
|  | *Actinobacillus succinogenes 130Z* | NC_009655.1 | 1 | 1 | 1 | 1 | 1 |
|  | *Haemophilus ducreyi 35000HP* | NC_002940.2 | 0 | 0 | 0 | 0 | 0 |
|  | *Haemophilus influenzae 86-028NP* | NC_007146.2 | 1 | 1 | 1 | 1 | 1 |
|  | *Haemophilus influenzae F3031* | NC_014920.1 | 0 | 0 | 0 | 0 | 0 |
|  | *Haemophilus influenzae F3047* | NC_014922.1 | 0 | 0 | 0 | 0 | 0 |
|  | *Haemophilus influenzae PittGG* | NC_009567.1 | 1 | 1 | 1 | 1 | 1 |
|  | *Haemophilus influenzae Rd KW20* | NC_000907.1 | 1 | 1 | 1 | 1 | 1 |
|  | *Haemophilus parainfluenzae T3T1* | NC_015964.1 | 1 | 1 | 1 | 1 | 1 |
|  | *Haemophilus parasuis SH0165* | NC_011852.1 | 1 | 1 | 1 | 1 | 1 |
|  | *Haemophilus somnus 129PT* | NC_008309.1 | 1 | 1 | 1 | 1 | 1 |
|  | *Haemophilus somnus 2336* | NC_010519.1 | 1 | 1 | 1 | 1 | 1 |
|  | *Mannheimia succiniciproducens MBEL55E* | NC_006300.1 | 1 | 1 | 1 | 1 | 1 |
|  | *Pasteurella multocida subsp. multocida str. Pm70* | NC_002663.1 | 1 | 1 | 1 | 1 | 1 |
| *Vibrionales* | *Photobacterium profundum SS9* | NC_006370.1, NC_006371.1 | 1 | 1 | 1 | 1 | 0 |
|  | *Vibrio anguillarum 775* | NC_015633.1, NC_015637.1 | 1 | 1 | 2 | 1 | 0 |
|  | *Vibrio cholerae M66-2* | NC_012578.1 | 1 | 1 | 2 | 1 | 0 |
|  | *Vibrio cholerae MJ-1236* | NC_012668.1 | 1 | 1 | 2 | 1 | 0 |
|  | *Vibrio cholerae O1 biovar El Tor str. N16961* | NC_002505.1 | 1 | 1 | 2 | 1 | 0 |
|  | *Vibrio cholerae O395* | NC_009457.1, NC_009456.1 | 1 | 1 | 2 | 1 | 0 |
|  | *Vibrio fischeri ES114* | NC_006840.2, NC_006841.2 | 1 | 1 | 1 | 1 | 0 |
|  | *Vibrio fischeri MJ11* | NC_011184.1, NC_011186.1 | 1 | 1 | 1 | 1 | 0 |
|  | *Vibrio harveyi ATCC BAA-1116* | NC_009783.1, NC_009784.1 | 1 | 1 | 2 | 1 | 0 |
|  | *Vibrio parahaemolyticus RIMD 2210633* | NC_004603.1, NC_004605.1 | 1 | 1 | 2 | 1 | 0 |
|  | *Vibrio sp. Ex25* | NC_013457.1, NC_013456.1 | 1 | 1 | 2 | 1 | 0 |
|  | *Vibrio splendidus LGP32* | NC_011753.2, NC_011744.2 | 1 | 1 | 2 | 1 | 0 |
|  | *Vibrio vulnificus CMCP6* | NC_004460.2, NC_004459.3 | 1 | 1 | 2 | 1 | 0 |
|  | *Vibrio vulnificus MO6-24/O* | NC_014965.1, NC_014966.1 | 1 | 1 | 2 | 1 | 0 |
|  | *Vibrio vulnificus YJ016* | NC_005139.1, NC_005140.1 | 1 | 1 | 2 | 1 | 0 |
| *Aeromonadales* | *Aeromonas hydrophila subsp. hydrophila ATCC 7966* | NC_008570.1 | 1 | 2 | 3 | 1 | 0 |
|  | *Aeromonas salmonicida subsp. salmonicida A449* | NC_009348.1 | 1 | 1 | 3 | 1 | 0 |
|  | *Tolumonas auensis DSM 9187* | NC_012691.1 | 1 | 2 | 2 | 1 | 0 |
| *Alteromonadales* | *Alteromonas macleodii str. 'Deep ecotype'* | NC_011138.3 | 1 | 1 | 1 | 1 | 1 |
|  | *Alteromonas sp. SN2* | NC_015554.1 | 1 | 1 | 2 | 1 | 1 |
|  | *Colwellia psychrerythraea 34H* | NC_003910.7 | 0 | 0 | 0 | 0 | 0 |
|  | *Ferrimonas balearica DSM 9799* | NC_014541.1 | 0 | 0 | 0 | 0 | 0 |
|  | *Idiomarina loihiensis L2TR* | NC_006512.1 | 0 | 0 | 0 | 0 | 0 |
|  | *Pseudoalteromonas atlantica T6c* | NC_008228.1 | 1 | 1 | 3 | 1 | 1 |
|  | *Pseudoalteromonas haloplanktis TAC125* | NC_007481.1, NC_007482.1 | 0 | 0 | 0 | 0 | 0 |
|  | *Pseudoalteromonas sp. SM9913* | NC_014803.1, NC_014800.1 | 1 | 1 | 1 | 1 | 1 |
|  | *Shewanella baltica OS155* | NC_009052.1 | 1 | 1 | 1 | 1 | 1 |
|  | *Shewanella baltica OS185* | NC_009665.1 | 1 | 1 | 1 | 1 | 1 |
|  | *Shewanella baltica OS195* | NC_009997.1 | 1 | 1 | 1 | 1 | 1 |
|  | *Shewanella baltica OS223* | NC_011663.1 | 1 | 1 | 1 | 1 | 1 |
|  | *Shewanella denitrificans OS217* | NC_007954.1 | 0 | 0 | 0 | 0 | 0 |
|  | *Shewanella frigidimarina NCIMB 400* | NC_008345.1 | 1 | 1 | 1 | 1 | 1 |
|  | *Shewanella halifaxensis HAW-EB4* | NC_010334.1 | 0 | 0 | 0 | 0 | 0 |
|  | *Shewanella loihica PV-4* | NC_009092.1 | 1 | 1 | 1 | 1 | 1 |
|  | *Shewanella oneidensis MR-1* | NC_004347.2 | 1 | 1 | 1 | 1 | 1 |
|  | *Shewanella pealeana ATCC 700345* | NC_009901.1 | 0 | 0 | 0 | 0 | 0 |
|  | *Shewanella piezotolerans WP3* | NC_011566.1 | 1 | 1 | 1 | 1 | 1 |
|  | *Shewanella putrefaciens CN-32* | NC_009438.1 | 1 | 1 | 1 | 1 | 1 |
|  | *Shewanella sediminis HAW-EB3* | NC_009831.1 | 0 | 0 | 0 | 0 | 0 |
|  | *Shewanella sp. ANA-3* | NC_008577.1 | 1 | 1 | 1 | 1 | 1 |
|  | *Shewanella sp. MR-4* | NC_008321.1 | 1 | 1 | 1 | 1 | 1 |
|  | *Shewanella sp. MR-7* | NC_008322.1 | 1 | 1 | 1 | 1 | 1 |
|  | *Shewanella sp. W3-18-1* | NC_008750.1 | 1 | 1 | 1 | 1 | 1 |
|  | *Shewanella violacea DSS12* | NC_014012.1 | 1 | 1 | 1 | 1 | 1 |
|  | *Shewanella woodyi ATCC 51908* | NC_010506.1 | 0 | 0 | 0 | 0 | 0 |
|  | *Psychromonas ingrahamii 37* | NC_008709.1 | 1 | 3 | 4 | 2 | 1 |
| *Pseudomonadales* | *Acinetobacter baumannii 1656-2* | NC_017162.1 | 0 | 0 | 0 | 0 | 0 |
|  | *Acinetobacter baumannii AB0057* | NC_011586.1 | 0 | 0 | 0 | 0 | 0 |
|  | *Acinetobacter baumannii AB307-0294* | NC_011595.1 | 0 | 0 | 0 | 0 | 0 |
|  | *Acinetobacter baumannii ACICU* | NC_010611.1 | 0 | 0 | 0 | 0 | 0 |
|  | *Acinetobacter baumannii ATCC 17978* | NC_009085.1 | 0 | 0 | 0 | 0 | 0 |
|  | *Acinetobacter baumannii AYE* | NC_010410.1 | 0 | 0 | 0 | 0 | 0 |
|  | *Acinetobacter baumannii SDF* | NC_010400.1 | 0 | 0 | 0 | 0 | 0 |
|  | *Acinetobacter baumannii TCDC-AB0715* | NC_017387.1 | 0 | 0 | 0 | 0 | 0 |
|  | *Acinetobacter calcoaceticus PHEA-2* | NC_016603.1 | 0 | 0 | 0 | 0 | 0 |
|  | *Acinetobacter sp. ADP1* | NC_005966.1 | 0 | 0 | 0 | 0 | 0 |
|  | *Acinetobacter sp. DR1* | NC_014259.1 | 0 | 0 | 0 | 0 | 0 |
|  | *Azotobacter vinelandii DJ* | NC_012560.1 | 1 | 1 | 0 | 1 | 1 |
|  | *Cellvibrio gilvus ATCC13127* | NC_010995.1 | 1 | 2 | 1 | 1 | 0 |
|  | *Moraxella catarrhalis RH4* | NC_014147.1 | 0 | 0 | 0 | 0 | 0 |
|  | *Pseudomonas aeruginosa LESB58* | NC_011770.1 | 1 | 1 | 0 | 1 | 1 |
|  | *Pseudomonas aeruginosa PA7* | NC_009656.1 | 1 | 1 | 0 | 1 | 1 |
|  | *Pseudomonas aeruginosa PAO1* | NC_002516.2 | 1 | 1 | 0 | 1 | 1 |
|  | *Pseudomonas aeruginosa UCBPP-PA14* | NC_008463.1 | 1 | 1 | 0 | 1 | 1 |
|  | *Pseudomonas brassicacearum subsp. brassicacearum NFM421* | NC_015379.1 | 1 | 1 | 0 | 1 | 1 |
|  | *Pseudomonas entomophila L48* | NC_008027.1 | 1 | 1 | 0 | 1 | 1 |
|  | *Pseudomonas fluorescens Pf-5* | NC_004129.6 | 1 | 1 | 0 | 1 | 1 |
|  | *Pseudomonas fluorescens Pf0-1* | NC_007492.2 | 1 | 1 | 0 | 1 | 1 |
|  | *Pseudomonas fluorescens SBW25* | NC_012660.1 | 1 | 1 | 0 | 1 | 1 |
|  | *Pseudomonas fulva 12-X* | NC_015556.1 | 1 | 1 | 0 | 1 | 1 |
|  | *Pseudomonas mendocina NK-01* | NC_015410.1 | 1 | 1 | 0 | 1 | 1 |
|  | *Pseudomonas mendocina ymp* | NC_009439.1 | 1 | 1 | 0 | 1 | 1 |
|  | *Pseudomonas putida F1* | NC_009512.1 | 1 | 1 | 0 | 1 | 1 |
|  | *Pseudomonas putida GB-1* | NC_010322.1 | 1 | 1 | 0 | 1 | 1 |
|  | *Pseudomonas putida KT2440* | NC_002947.3 | 1 | 1 | 0 | 1 | 1 |
|  | *Pseudomonas putida S16* | NC_015733.1 | 1 | 1 | 0 | 1 | 1 |
|  | *Pseudomonas putida W619* | NC_010501.1 | 1 | 1 | 0 | 1 | 1 |
|  | *Pseudomonas stutzeri A1501* | NC_009434.1 | 1 | 1 | 0 | 1 | 1 |
|  | *Pseudomonas stutzeri ATCC 17588 = LMG 11199* | NC_015740.1 | 1 | 1 | 0 | 1 | 1 |
|  | *Pseudomonas syringae pv. phaseolicola 1448A* | NC_005773.3 | 1 | 1 | 0 | 1 | 1 |
|  | *Pseudomonas syringae pv. syringae B728a* | NC_007005.1 | 1 | 1 | 0 | 1 | 1 |
|  | *Pseudomonas syringae pv. tomato str. DC3000* | NC_004578.1 | 1 | 1 | 0 | 1 | 1 |
|  | *Psychrobacter sp. PRwf-1* | NC_009524.1 | 0 | 0 | 0 | 0 | 0 |
| *Oceanospirillales* | *Alcanivorax borkumensis SK2* | NC_008260.1 | 0 | 0 | 0 | 0 | 0 |
|  | *Hahella chejuensis* KCTC2396 | NC_007645.1 | 0 | 0 | 0 | 0 | 0 |
|  | *Halomonas elongata DSM2581* | NC_014532.1 | 0 | 0 | 0 | 0 | 0 |
|  | *Chromohalobacter salexigens* DSM3043 | NC_007963.1 | 0 | 0 | 0 | 0 | 0 |
| *Xanthomonadales* | *Pseudoxanthomonas suwonensis 11-1* | NC_014924.1 | 0 | 0 | 0 | 0 | 0 |
|  | *Stenotrophomonas maltophilia K279a* | NC_010943.1 | 1 | 1 | 0 | 1 | 0 |
|  | *Stenotrophomonas maltophilia R551-3* | NC_011071.1 | 0 | 0 | 0 | 0 | 0 |
|  | *Xanthomonas axonopodis pv. citri str. 306* | NC_003919.1 | 2 | 2 | 0 | 1 | 0 |
|  | *Xanthomonas campestris pv. campestris str. 8004* | NC_007086.1 | 2 | 2 | 0 | 1 | 0 |
|  | *Xanthomonas campestris pv. campestris str. ATCC 33913* | NC_003902.1 | 2 | 2 | 0 | 1 | 0 |
|  | *Xanthomonas campestris pv. campestris str. B100* | NC_010688.1 | 2 | 2 | 0 | 1 | 0 |
|  | *Xanthomonas campestris pv. vesicatoria str. 85-10* | NC_007508.1 | 2 | 2 | 0 | 1 | 0 |
|  | *Xanthomonas oryzae pv. oryzae KACC10331* | NC_006834.1 | 2 | 2 | 0 | 1 | 0 |
|  | *Xanthomonas oryzae pv. oryzae MAFF 311018* | NC_007705.1 | 2 | 2 | 0 | 1 | 0 |
|  | *Xanthomonas oryzae pv. oryzae PXO99A* | NC_010717.1 | 2 | 2 | 0 | 1 | 0 |
|  | *Xylella fastidiosa 9a5c* | NC_002488.3 | 0 | 0 | 0 | 0 | 0 |
|  | *Xylella fastidiosa M12* | NC_010513.1 | 0 | 0 | 0 | 0 | 0 |
|  | *Xylella fastidiosa M23* | NC_010577.1 | 0 | 0 | 0 | 0 | 0 |
|  | *Xylella fastidiosa Temecula1* | NC_004556.1 | 0 | 0 | 0 | 0 | 0 |
| *Methylococcales* | *Methylomonas methanica MC09* | NC_015572.1 | 2 | 1 | 1 | 1 | 1 |
|  | *Methylococcus capsulatus str. Bath* | NC_002977.6 | 1 | 1 | 1 | 2 | 2 |
| *Chromatiales* | *Allochromatium vinosum DSM 180* | NC_013851.1 | 1 | 2 | 2 | 3 | 2 |
|  | *Nitrosococcus halophilus Nc4* | NC_013960.1 | 2 | 2 | 1 | 2 | 1 |
|  | *Nitrosococcus oceani ATCC 19707* | NC_007484.1 | 1 | 1 | 1 | 2 | 1 |
|  | *Nitrosococcus watsonii C-113* | NC_014315.1 | 1 | 1 | 1 | 2 | 1 |
|  | *Halorhodospira halophila SL1* | NC_008789.1 | 1 | 0 | 2 | 1 | 1 |
|  | *Thioalkalivibrio sp. K90mix* | NC_013889.1 | 1 | 0 | 1 | 1 | 1 |
|  | *Thioalkalivibrio sulfidophilus HL-EbGr7* | NC_011901.1 | 1 | 1 | 1 | 1 | 1 |
| *Cardiobacteriales* | *Dichelobacter nodosus VCS1703A* | NC_009446.1 | 0 | 0 | 0 | 0 | 0 |
| *Legionelalles* | *Coxiella burnetii CbuG_Q212* | NC_011527.1 | 0 | 0 | 0 | 0 | 0 |
|  | *Coxiella burnetii CbuK_Q154* | NC_011528.1 | 0 | 0 | 0 | 0 | 0 |
|  | *Coxiella burnetii Dugway 5J108-111* | NC_009727.1 | 0 | 0 | 0 | 0 | 0 |
|  | *Coxiella burnetii RSA 331* | NC_010117.1 | 0 | 0 | 0 | 0 | 0 |
|  | *Coxiella burnetii RSA 493* | NC_002971.3 | 0 | 0 | 0 | 0 | 0 |
|  | *Legionella longbeachae NSW150* | NC_013861.1 | 0 | 0 | 0 | 0 | 0 |
|  | *Legionella pneumophila 2300/99 Alcoy* | NC_014125.1 | 0 | 0 | 0 | 0 | 0 |
|  | *Legionella pneumophila str. Corby* | NC_009494.2 | 0 | 0 | 0 | 0 | 0 |
|  | *Legionella pneumophila str. Lens* | NC_006369.1 | 0 | 0 | 0 | 0 | 0 |
|  | *Legionella pneumophila str. Paris* | NC_006368.1 | 0 | 0 | 0 | 0 | 0 |
|  | *Legionella pneumophila subsp. pneumophila str. Philadelphia 1* | NC_002942.5 | 0 | 0 | 0 | 0 | 0 |
| *Thiotricales* | *Francisella novicida U112* | NC_008601.1 | 1 | 0 | 1 | 1 | 1 |
|  | *Francisella philomiragia subsp. philomiragia ATCC 25017* | NC_010336.1 | 1 | 0 | 1 | 1 | 1 |
|  | *Francisella sp. TX077308* | NC_015696.1 | 1 | 0 | 1 | 1 | 1 |
|  | *Francisella tularensis subsp. holarctica FTNF002-00* | NC_009749.1 | 1 | 0 | 1 | 1 | 1 |
|  | *Francisella tularensis subsp. holarctica LVS* | NC_007880.1 | 1 | 0 | 1 | 1 | 1 |
|  | *Francisella tularensis subsp. holarctica OSU18* | NC_008369.1 | 1 | 0 | 1 | 1 | 1 |
|  | *Francisella tularensis subsp. mediasiatica FSC147* | NC_010677.1 | 1 | 0 | 1 | 1 | 1 |
|  | *Francisella tularensis subsp. tularensis WY96-3418* | NC_009257.1 | 1 | 0 | 1 | 1 | 1 |
