# Supplementary material for: Comparative Genomic and Phylogenetic Analyses of Gammaproteobacterial glg Genes Traced the Origin of the Escherichia coli Glycogen glgBXCAP Operon to the Last Common Ancestor of the Sister Orders Enterobacteriales and Pasteurellales
Source: PLoS One. 2015 Jan 21;10(1):e0115516. doi: 10.1371/journal.pone.0115516 (PMC4301808; doi:10.1371/journal.pone.0115516)
Supplement: S2 Table — (DOCX) [file pone.0115516.s002.docx]

**Table S2**

| **Bacterial group** | **Species** | **Genome accession number** | **Copy number** | | | | |
| --- | --- | --- | --- | --- | --- | --- | --- |
|  |  |  | ***glgB*** | ***glgX*** | ***glgC*** | ***glgA*** | ***glgP*** |
| *Acidithiobacillales* | *Acidithiobacillus caldus SM-1* | NC_015850.1 | 1 | 1 | 1 | 1 | 1 |
|  | *Acidithiobacillus ferrivorans SS3* | NC_015942.1 | 1 | 1 | 1 | 1 | 1 |
|  | *Acidithiobacillus ferrooxidans ATCC 23270* | NC_011761.1 | 1 | 0 | 1 | 1 | 1 |
| *Betaproteobacteria* | *Bordetella parapertussis 12822* | NC_002928.3 | 1 | 0 | 0 | 1 | 0 |
|  | *Burkholderia sp. 383* | NC_007510.1, NC_007511.1, NC_007509.1 | 1 | 1 | 0 | 0 | 0 |
|  | *Leptothrix cholodnii SP-6* | NC_010524.1 | 1 | 1 | 1 | 1 | 1 |
|  | *Methylovorus glucosetrophus SIP34* | NC_012969.1 | 1 | 1 | 1 | 1 | 1 |
|  | *Nitrosomonas europaea ATCC 19718* | NC_004757.1 | 1 | 0 | 1 | 1 | 0 |
|  | *Nitrosospira multiformis ATCC 25196* | NC_007614.1 | 1 | 1 | 1 | 1 | 0 |
|  | *Ralstonia eutropha H16* | NC_008313.1, NC_008314.1 | 1 | 0 | 0 | 0 | 1 |
|  | *Thiomonas intermedia K12* | NC_014153.1 | 1 | 1 | 1 | 1 | 1 |
|  | *Thauera sp. MZ1T* | NC_011662.2 | 1 | 1 | 1 | 1 | 1 |
|  | *Thiobacillus denitrificans ATCC 25259* | NC_007404.1 | 1 | 0 | 1 | 1 | 1 |
|  | *Variovorax paradoxus S110* | NC_012791.1 | 2 | 2 | 1 | 1 | 1 |
| *Alphaproteobacteria* | *Acetobacter pasteurianus IFO 3283-01* | NC_013209.1 | 2 | 2 | 0 | 2 | 0 |
|  | *Agrobacterium tumefaciens C58* | NC_003063.2 | 1 | 1 | 1 | 1 | 1 |
|  | *Azospirillum sp. B510* | NC_013854.1 | 1 | 1 | 1 | 1 | 1 |
|  | *Mesorhizobium loti MAFF303099* | NC_002678.2 | 1 | 0 | 1 | 1 | 1 |
|  | *Methylobacterium nodulans ORS 2060* | NC_011894.1 | 1 | 2 | 0 | 1 | 1 |
|  | *Rhizobium tropici CIAT 899* | NC_020059.1 | 1 | 1 | 1 | 1 | 1 |
|  | *Rhodobacter sphaeroides ATCC 17029* | NC_009049.1, NC_009050.1 | 1 | 3 | 1 | 1 | 1 |
|  | *Rhodospirillum centenum SW* | NC_011420.2 | 2 | 2 | 1 | 1 | 1 |
|  | *Sinorhizobium fredii USDA 257* | NC_018000.1 | 1 | 1 | 1 | 2 | 1 |
| *Deltaproteobacteria* | *Desulfovibrio vulgaris str. Hildenborough* | NC_002937.3 | 1 | 0 | 0 | 1 | 0 |
|  | *Myxococcus fulvus HW-1* | NC_015711.1 | 1 | 2 | 1 | 1 | 1 |
|  | *Stigmatella aurantiaca DW4/3-1* | NC_014623.1 | 1 | 2 | 2 | 1 | 1 |
|  | *Sorangium cellulosum So Ce 56* | NC_010162.1 | 1 | 1 | 2 | 1 | 1 |
| *Firmicutes* | *Acetobacterium woodii DSM 1030* | NC_016894.1 | 1 | 0 | 0 | 1 | 2 |
|  | *Bacillus licheniformis DSM13=ATCC14580* | NC_006270.3 | 1 | 0 | 1 | 1 | 1 |
|  | *Bacillus megaterium DSM 319* | NC_014103.1 | 1 | 0 | 1 | 1 | 1 |
|  | *Bacillus subtilis subsp. subtilis str. 168* | NC_000964.3 | 1 | 0 | 1 | 1 | 1 |
|  | *Clostridium acidurici 9a* | NC_018664.1 | 1 | 0 | 1 | 1 | 1 |
|  | *Clostridium perfringens str. 13* | NC_003366.1 | 2 | 0 | 1 | 1 | 2 |
|  | *Clostridium thermocellum ATCC 27405* | NC_009012.1 | 1 | 0 | 1 | 2 | 0 |
|  | *Geobacillus sp. Y412MC52* | NC_014915.1 | 1 | 0 | 0 | 1 | 1 |
|  | *Lactobacillus plantarum WCFS1* | NC_004567.2 | 1 | 0 | 1 | 1 | 1 |
|  | *Streptococcus mutans NN2025* | NC_013928.1 | 1 | 0 | 1 | 1 | 0 |
|  | *Streptococcus pneumoniae D39* | NC_008533.1 | 1 | 0 | 1 | 1 | 0 |
| *Spirochaetales* | *Brachyspira hyodysenteriae WA1* | NC_012225.1 | 0 | 0 | 1 | 1 | 0 |
|  | *Leptospira biflexa serovar Patoc (Paris)* | NC_010602.1, NC_010843.1 | 0 | 0 | 1 | 1 | 1 |
|  | *Spirochaeta africana Z-7692* | NC_017098.1 | 0 | 2 | 1 | 1 | 1 |
|  | *Treponema denticola ATCC 35405* | NC_002967.9 | 0 | 0 | 1 | 1 | 1 |
| *Chlamydiales* | *Candidatus Protochlamydia amoebophila UWE25* | NC_005861.1 | 1 | 1 | 1 | 1 | 1 |
|  | *Chamydia muridarum Nigg* | NC_002620.2 | 1 | 1 | 1 | 1 | 1 |
|  | *Chlamydia trachomatis D/UW-3/CX* | NC_000117.1 | 1 | 1 | 1 | 1 | 1 |
|  | *Chlamydophila abortus S26/3* | NC_004552.2 | 1 | 1 | 1 | 1 | 1 |
|  | *Chlamydophila caviae GPIC* | NC_003361.3 | 1 | 1 | 1 | 1 | 1 |
|  | *Chlamydophila pneumoniae CWL029* | NC_000922.1 | 1 | 1 | 1 | 1 | 1 |
|  | *Opitutus terrae PB90-1* | NC_010571.1 | 1 | 1 | 1 | 1 | 1 |
| *Chloroby/* | *Chlorobium tepidum TLS* | NC_002932.3 | 0 | 0 | 0 | 1 | 0 |
| *Bacteroidetes* | *Gramella forsetii KT0803* | NC_008571.1 | 1 | 0 | 0 | 1 | 0 |
|  | *Maribacter sp. HTCC2170* | NC_014472.1 | 1 | 0 | 0 | 0 | 0 |
|  | *Pedobacter heparinus DSM 2366* | NC_013061.1 | 1 | 0 | 1 | 1 | 0 |
|  | *Polaribacter sp. MED152* | NC_020830.1 | 1 | 0 | 1 | 0 | 0 |
|  | *Salinibacter ruber DSM 13855* | NC_007677.1 | 1 | 1 | 1 | 0 | 0 |
| *Actinobacteria* | *Bifidobacterium longum NCC2705* | NC_004307.2 | 1 | 1 | 1 | 0 | 1 |
|  | *Corynebacterium diphtheriae NCTC13129* | NC_002935.2 | 1 | 1 | 1 | 0 | 1 |
|  | *Leifsonia xyli subsp. Xyli str. CTCB07* | NC_006087.1 | 1 | 0 | 1 | 0 | 0 |
|  | *Mycobacterium avium subsp. Paratuberculosis str. K10* | NC_002944.2 | 1 | 2 | 1 | 0 | 0 |
|  | *Mycobacterium bovis AF2122/97* | NC_002945.3 | 1 | 0 | 1 | 0 | 0 |
|  | *Mycobacterium tuberculosis H37RV* | NC_000962.3 | 1 | 0 | 1 | 0 | 0 |
|  | *Nocardia farcinica IFM 10152* | NC_006361.1 | 1 | 1 | 1 | 0 | 0 |
|  | *Propionibacterium acnes KPA171202* | NC_006085.1 | 1 | 1 | 1 | 0 | 1 |
|  | *Streptomyces avermitilis MA-4680* | NC_003155.4 | 2 | 2 | 1 | 0 | 0 |
|  | *Streptomyces coelicolor A3(2)* | NC_003888.3 | 2 | 3 | 1 | 0 | 0 |
| *Cyanobacteria* | *Acarychloris marina MBIC11017* | NC_009925.1 | 1 | 0 | 2 | 2 | 1 |
|  | *Anabaena variabilis ATCC 29413* | NC_007413.1 | 1 | 0 | 1 | 1 | 0 |
|  | *Cyanothece sp. PCC 8802* | NC_013161.1 | 2 | 3 | 1 | 2 | 1 |
|  | *Gloeobacter violaceus PCC7421* | NC_005125.1 | 1 | 0 | 1 | 1 | 1 |
|  | *Microcystis aeruginosa NIES-843* | NC_010296.1 | 1 | 1 | 1 | 2 | 1 |
|  | *Nostoc sp. PCC7120* | NC_003272.1 | 1 | 0 | 1 | 2 | 0 |
|  | *Prochlorococcus marinus str. MIT9301* | NC_009091.1 | 1 | 1 | 1 | 1 | 1 |
|  | *Synechococcus elongatus PCC 6301* | NC_006576.1 | 1 | 1 | 1 | 1 | 1 |
|  | *Synechocystis sp. PCC 6803 substr. PCC-N* | NC_017052.1 | 1 | 2 | 1 | 2 | 2 |
